# Impact of ATF6 deletion on the embryonic brain development

**DOI:** 10.1016/j.isci.2025.112569

**Published:** 2025-05-02

**Authors:** Loc Dinh Nguyen, Ly Huong Nguyen, Dat Xuan Dao, Tsuyoshi Hattori, Mika Takarada-Iemata, Hiroshi Ishii, Takashi Tamatani, Hiroshi Kawasaki, Yohei Shinmyo, Kenta Onoue, Shigenobu Yonemura, Jun Zhang, Masato Miyake, Seiichi Oyadomari, Kazutoshi Mori, Osamu Hori

**Affiliations:** 1 Department of Neuroanatomy, Graduate School of Medical Sciences, Kanazawa University, Kanazawa, Ishikawa, Japan; 2 Department of Medical Neuroscience, Graduate School of Medical Sciences, Kanazawa University, Kanazawa, Ishikawa, Japan; 3 Department of Neurophysiology, Hamamatsu University School of Medicine, Hamamatsu, Shizuoka, Japan; 4 Laboratory for Ultrastructural Research, RIKEN Center for Biosystems Dynamics Research, Kobe, Hyogo, Japan; 5 Department of Cell Biology, Tokushima University Graduate School of Medicine, Tokushima, Japan; 6 Division of Molecular Biology, Institute of Advanced Medical Sciences, Tokushima University, Tokushima, Japan; 7 Kyoto University Institute for Advanced Study, Kyoto, Japan

**Keywords:** Neuroscience, Cell biology, Developmental biology

## Abstract

Although the unfolded protein response (UPR) is activated during brain development, its roles remain unclear. Here, we report that deletion of activating transcription factor 6 (ATF6), consisting of ATF6α and ATF6β, in the developing brain caused microcephaly and neonatal death in mice. Analysis of Atf6a/Atf6b double conditional knockout (dcKO) brains revealed diverse neuronal phenotypes, such as reduced neurogenesis, increased cell death, impaired cortical layer formation, and axon projection defects. Furthermore, hypervasculature, glial defects, and neuroinflammation were observed in dcKO brains. Notably, hypervasculature was detected at E14.5, when endoplasmic reticulum (ER) stress was morphologically unclear, but the UPR was activated to a greater extent in dcKO brains. Expression profiles revealed reduced levels of molecular chaperones in the ER and enhanced levels of PERK- and IRE1-downstream molecules, including VEGFA, in dcKO brains. Administration of a chemical chaperone 4-phenylbutyric acid partially rescued dcKO mice, suggesting roles of ATF6 for improving proteostasis and for coordinating the UPR.

## Highlights

• Deletion of ATF6α and ATF6β caused microcephaly and neonatal death in mice
• Deletion of ATF6α and ATF6β impaired neuronal and glial development
• A chemical chaperone 4-PBA partially rescued ATF6α- and ATF6β-deleted mice

## Graphical abstract

**Figure:**
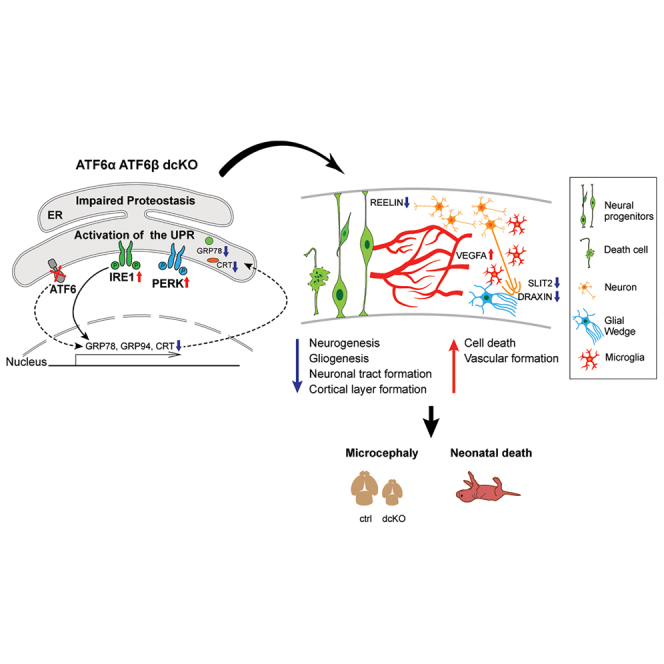

## Introduction

To complete a variety of functions, such as the synthesis of secretory proteins and lipids and storage of intracellular Ca^2+^ in different physiological settings, the endoplasmic reticulum (ER) has developed a system that senses and responds to environmental changes in cells. When cells are subjected to challenges, such as energy shortage, impaired Ca^2+^ homeostasis, and increased protein synthesis, unfolded proteins accumulate in the ER, leading to a condition, generally termed ER stress. Cells respond to ER stress by activating the unfolded protein response (UPR), which is mediated by at least three ER-resident membrane proteins: protein kinase R (PKR)-like ER kinase (PERK), inositol-requiring enzyme 1 (IRE1), and activating transcription factor 6 (ATF6).^1^^,^^2^ The UPR usually adapts to environmental changes, but it may also cause cell death if the level of ER stress exceeds the folding capacity of the ER or if there are defects in the UPR. In the central nervous system (CNS), pathological conditions, such as neurodegeneration, brain ischemia, excitotoxicity, and neuroinflammation, are associated with ER stress.^3^^,^^4^^,^^5^ Furthermore, ER stress and UPR may play critical roles in the development of brain.^6^^,^^7^^,^^8^

Among UPR transducers, ATF6 is responsible for the induction of major molecular chaperones in the ER, such as glucose-regulated protein 78 (GRP78), glucose-regulated protein 94 (GRP94), and calreticulin (CRT), as well as several ER-associated degradation components. In mammals, there are two subtypes of ATF6, ATF6α and ATF6β. Although ATF6α plays a dominant role in the transcriptional activation in response to ER stress,^9^ ATF6β is also ubiquitously expressed in tissues including brain. In addition to the roles of ATF6α in the neuropathological conditions,^10^^,^^11^^,^^12^ we recently reported critical roles of ATF6β in Ca^2+^ homeostasis and survival of neurons under ER stress^13^^,^^14^ and in the adaptation to the psychiatric conditions.^15^ To meet the increased demands in the ER, ATF6α/β-mediated adjustment of chaperone levels was essential for the development of the notochord, a transient rod-like structure at the developing midline in embryos.^9^^,^^16^

To overcome the early embryonic lethality of *Atf6a*^−/−^*Atf6b*^−/−^ mice^9^ and to dissect the roles of ATF6 branch in the CNS, we developed NESTIN-driven *Atf6α/Atf6b* double conditional knockout (dcKO) mice (*Nes-Cre Atf6a*^*fl/fl*^*Atf6b*^*fl/fl*^ mice). Here, we report that deletion of ATF6 in the developing brain causes microcephaly and neonatal death, the latter is associated with impaired milk suckling but not with impaired respiration. Further analyses revealed reduced proliferation and increased death of progenitors, impaired cortical layer formation, axon projection defects, glial defects, hypervasculature, and neuroinflammation. Together with the expression profile results, we propose that ATF6 deletion hyperactivates the PERK and IRE1 branches of the UPR in addition to impairing proteostasis, and dysregulates the microenvironment required for brain development in mice.

## Results

### Expressions of ATF6α, ATF6β, and other UPR genes in the developing brains

**Figure 1:**
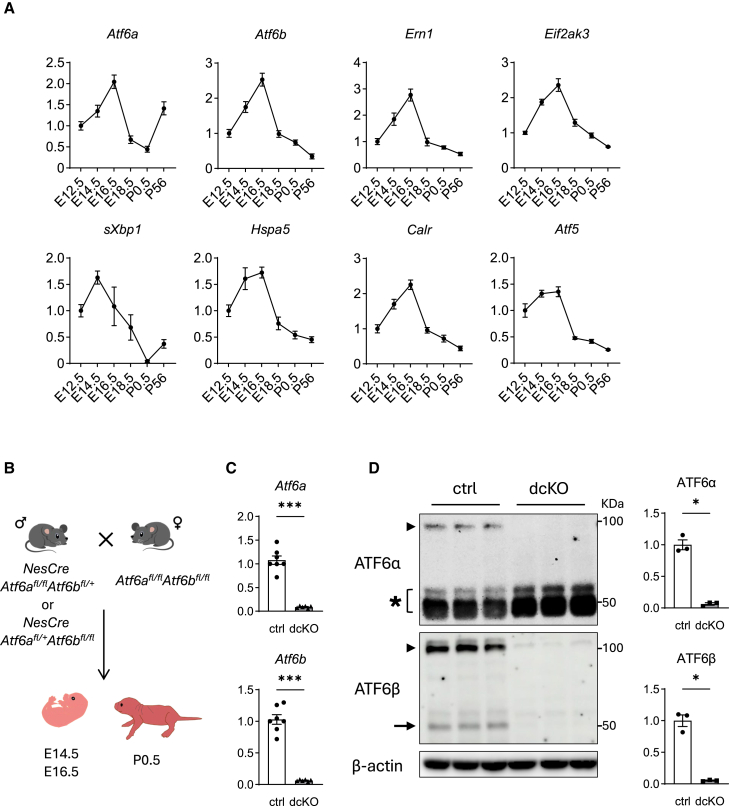
Expression of UPR genes in developing brain (A) Expression of UPR genes in wild-type cerebral cortex at different stages of development (*n* = 4 brains per time point), data are shown as mean ± SEM. E12.5, E14.5, E16.5, and E18.5 denote embryonic 12.5, 14.5, 16.5, and 18.5 days after gestation, respectively. P0.5 and P56 denote postnatal day 0.5 and 56, respectively. (B) Crossing scheme for parental mating, embryos or neonatal mice were used for experiments after genotyping. (C and D) Expression of *Atf6a* and *Atf6b* mRNA (C) (*n* = 6–7 brains per group) and protein (D) (*n* = 3 brains per group) in E16.5 cerebral cortices from control and dcKO embryos. Data are shown as mean ± SEM. ∗*p* < 0.05 and ∗∗∗*p* < 0.001 by Mann-Whitney U test. Arrowhead: full length ATF6α and ATF6β, arrow: N-terminal fragment, asterisk: non-specific band.

Although UPR activation has been reported in the developing brain,^17^ its involvement with the ATF6 branch remains unclear. Therefore, we first examined expressions of ATF6α, ATF6β, and other UPR genes in the developing brain. Quantitative reverse transcription polymerase chain reaction (RT-qPCR) revealed that both of *Atf6α* and *Atf6b*, and *Ern1* (IRE1a) and *Eif2ak3* (PERK) mRNAs increased and peaked at embryonic 16.5 days after gestation (E16.5) (Figure 1A). The downstream target genes of the ATF6 branch, such as *Hspa5* (GRP78) and *Calr* (CRT) showed similar expression patterns, although the former had a slightly higher expression level at E14.5 (Figure 1A). This may be due to the effect of the IRE1 branch, as peak expression of *sXbp1* (spliced form of X-box binding protein 1) was observed at E14.5. The expressions of PERK target genes such as *Atf4* (data not shown) and *Atf5* (Figure 1A) was also high at E14.5–E16.5. After birth, expression of *Atf6α*, but not *Atf6b*, increased again at postnatal day 56 (P56).

### Construction of Atf6α/Atf6b dcKO mice (Nes-Cre Atf6a^fl/fl^Atf6b^fl/fl^ mice)

To study the role of the ATF6 branch in the developing brain, *Atf6a*^*fl/fl*^*Atf6b*^*fl/fl*^ mice were generated by crossing *Atf6a*^*fl/fl*^ mice^18^ and *Atf6b*^*fl/fl*^ mice, the latter was designed and developed, as described in the STAR Methods and in Figure S1. *Atf6a*^*fl/fl*^*Atf6b*^*fl/fl*^ female mice were further crossed with *Nes*-*Cre Atf6a*^*fl/+*^*Atf6b*^*fl/fl*^ or *Nes-Cre Atf6a*^*fl/fl*^*Atf6b*^*fl/+*^ male mice to establish *Nes-Cre Atf6a*^*fl/fl*^*Atf6b*^*fl/fl*^ mice.

To confirm the expression of ATF6α and ATF6β in the control (*Atf6a*^*fl/fl*^*Atf6b*^*fl/fl*^) brains and the deletion of both genes in dcKO(*Nes-*Cre *Atf6a*^*fl/fl*^*Atf6b*^*fl/fl*^) brains, RNA and protein extracts from E16.5 cerebral cortices were subjected to RT-qPCR and western blot, respectively. RT-qPCR revealed almost no expression of *Atf6α* or *Atf6b* in dcKO brains (Figure 1C). Consistently, western blot revealed full lengths of ATF6α and ATF6β proteins in control brains but not in dcKO brains (Figure 1D). Furthermore, N-terminal fragment (NTF: active form) of ATF6β, was detected in control brains, but not in dcKO brains. The NTF of ATF6α was unclear because of the thick non-specific band (Figure 1D).

### Neonatal death and microcephaly in dcKO mice

**Figure 2:**
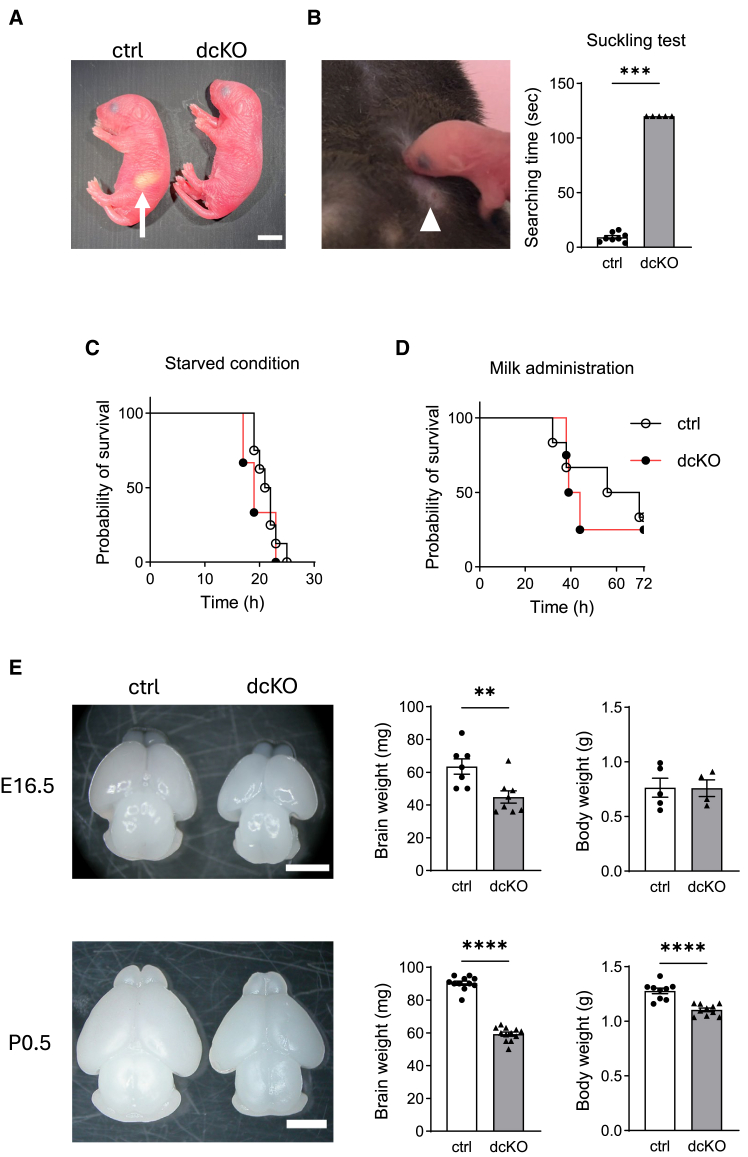
Behavior and brain size of the control and dcKO mice (A) Newborn mice at P0.5 in the same littermate. Arrow indicates a milk spot. (B) Illustration of suckling behavior test. Nipple searching time is shown in the graph as mean ± SEM (*n* = 8 for control mice including 4 *Atf6a*^*fl/fl*^*Atf6b*^*fl/fl*^ mice and 4 *Atf6a*^*fl/fl*^*Atf6b*^*fl/+*^ mice, *n* = 5 for dcKO mice). All dcKO mice failed to find and attach to the nipple (arrowhead) after 120 s and were considered negative in this test. (C and D) Neonatal mice after caesarean delivery were monitored in warm-humidified chamber either without feeding (*n* = 8 for control mice, *n* = 3 for dcKO mice, *p* = 0.5093 by Mantel-Cox test) (C) or with administration of the artificial milk directly into the stomach (*n* = 6 for control mice, *n* = 4 for dcKO mice, *p* = 0.6822 by Mantel-Cox test). (E) Brains from E16.5 embryos and P0.5 mice. Brain and body weights were measured at both E16.5 (*n* = 4–8 embryos for each group) and P0.5 (*n* = 9–12 mice for each group). Data are represented as mean ± SEM. ∗∗*p* < 0.01, ∗∗∗*p* < 0.001, and ∗∗∗∗*p* < 0.0001 by Mann-Whitney U test. Scale bars: 5 mm (A), 2 mm (E).

Although dcKO mice were born at an almost Mendelian ratio (Figure S2A), they died within 24 h of birth. The skin was pink, and the limbs moved almost normally. However, milk spots, which represent the storage of milk in the stomach (Figure 2A, arrow), were not observed in most cases (Figure 2A), and the stomach was empty (Figure S2B). Although previous reports have described respiratory failure-associated neonatal death in two systemic GRP78-mutant mice,^6^^,^^7^ gross observations and hematoxylin and eosin (H&E) staining revealed that the lungs of dcKO mice expanded well and showed a morphology similar to that of control mice (Figures S2C and S2D). These results suggest that suckling failure is the primary cause of neonatal death in dcKO mice. Therefore, suckling test was performed, as described previously^19^ (Figure 2B). After placing the mouth close to the mother’s nipple, control pups soon started to suckle milk, but dcKO mice did not start within 120 s. Importantly, the dcKO mice did not suckle even when putting their mouths directly on mother’s nipple. To confirm the critical role of suckling in dcKO mice, Caesarean section-derived neonates were monitored either without feeding (Figure 2C) or with administration of artificial milk directly into the stomach (Figure 2D), as described previously.^20^ The control and dcKO mice showed similar survival rates in both conditions.

As suckling defects can be associated with impaired neuronal function,^19^ the brains of the two genotypes were macroscopically compared. The brains of dcKO mice were smaller than those of control mice both at E16.5 and P0.5 (Figure 2E, left graphs). In contrast, the body weights were similar at E16.5, while they were lower in dcKO mice at P0.5 (Figure 2E, right graphs), which may be due to impaired milk suckling.

Mice with other genotypes, such as *Nes*-*Cre Atf6a*^*fl/+*^*Atf6b*^*fl/fl*^, *Nes-Cre Atf6a*^*fl/fl*^*Atf6b*^*fl/+*^, *Atf6a*^*fl/fl*^*Atf6b*^*fl/+*^, and *Atf6a*^*fl/+*^*Atf6b*^*fl/fl*^ developed normally, suckled milk well, and their brains sizes were similar to those of control and wild-type C57BL/6N mice. Therefore, further experiments were performed using the dcKO and control mice.

### Reduced neurogenesis and enhanced cell death in dcKO mice

**Figure 3:**
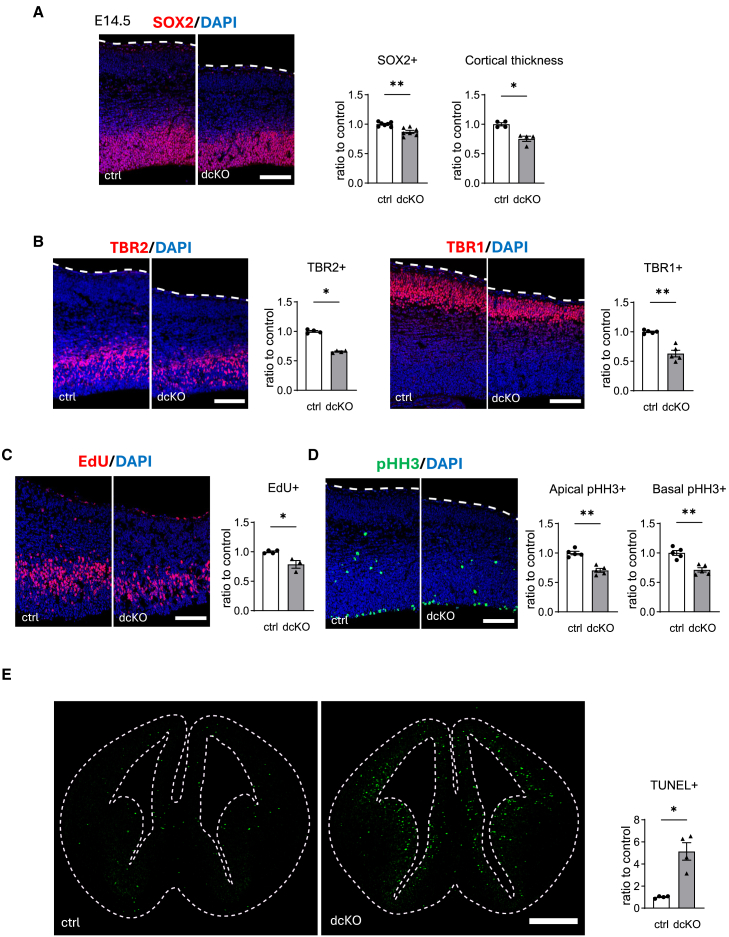
Impaired neurogenesis and increased cell death in dcKO (A, B, and D) Immunohistochemistry of the brain section from E14.5 control or dcKO embryos for the indicated molecules. Cortical thickness was also measured by the thickness of DAPI positive area (A) (*n* = 4–7 brains for each group). Dashed lines indicate brain surface. (C) EdU incorporation was conducted at E14.5 for 1 h to see proliferating cells in S-phase (EdU) (4 control brains including 2 *Atf6a*^*fl/fl*^*Atf6b*^*fl/fl*^ embryos and 2 *Atf6a*^*fl/fl*^*Atf6b*^*fl/+*^ embryos, and 4 dcKO brains). (E) Apoptosis was evaluated by TUNEL staining using sections prepared for immunohistochemistry as aforementioned (*n* = 4 brains for each group). Dashed lines indicate brain surface and lateral ventricle. Data are represented as mean ± SEM. ∗*p* < 0.05 and ∗∗*p* < 0.01 by Mann-Whitney U test. Scale bars: 100 μm (A–D), 500 μm (E).

As enhanced level of ER stress and prolonged activation of the UPR, especially that of PERK branch, disturb the proliferation of intermediate progenitor cells (IP), leading to microcephaly,^8^ the status of neurogenesis and cell death was analyzed in each genotype at E14.5. The thickness of the cerebral cortex was reduced in dcKO mice, which was consistent with the small size of the brain (Figure 3A). The numbers of sex-determining region Y (SRY)-box 2 (SOX2)(+) neural stem cells/apical progenitor cells (AP) (Figure 3A), T-box brain protein 2 (TBR2)(+) IP (Figure 3B, left), and T-box brain protein 1 (TBR1)(+) mature neurons (Figure 3B, right) were all reduced in dcKO mice. Next, proliferation status was analyzed using 5-ethynyl-2′-deoxyuridine (EdU) incorporation assay (S phase) and immunohistochemistry for phospho-histone H3 (pHH3) (M phase). The number of EdU-incorporated and pHH3(+) cells was reduced in dcKO mice (Figures 3C and 3D). The status of cell death was also analyzed under the same conditions using the terminal deoxynucleotidyl transferase dUTP nick end labeling (TUNEL) assay. Higher numbers of TUNEL(+) cells were observed in the regions surrounding the lateral ventricles of dcKO mice (Figure 3E).

### Hypervasculature in dcKO brains

To determine more detail of the morphological changes in dcKO mice, E14.5 brain samples were prepared for electron microscopy (EM). Analysis of wide-area photos of the cerebral cortices revealed more vessels in dcKO mice (Figure S3A). The vessel lumens were morphologically similar between the two genotypes (Figure S3A). In contrast, analysis at higher magnification did not show clear differences in the structure of organelles including the ER between the two genotypes (Figure S3B).

**Figure 4:**
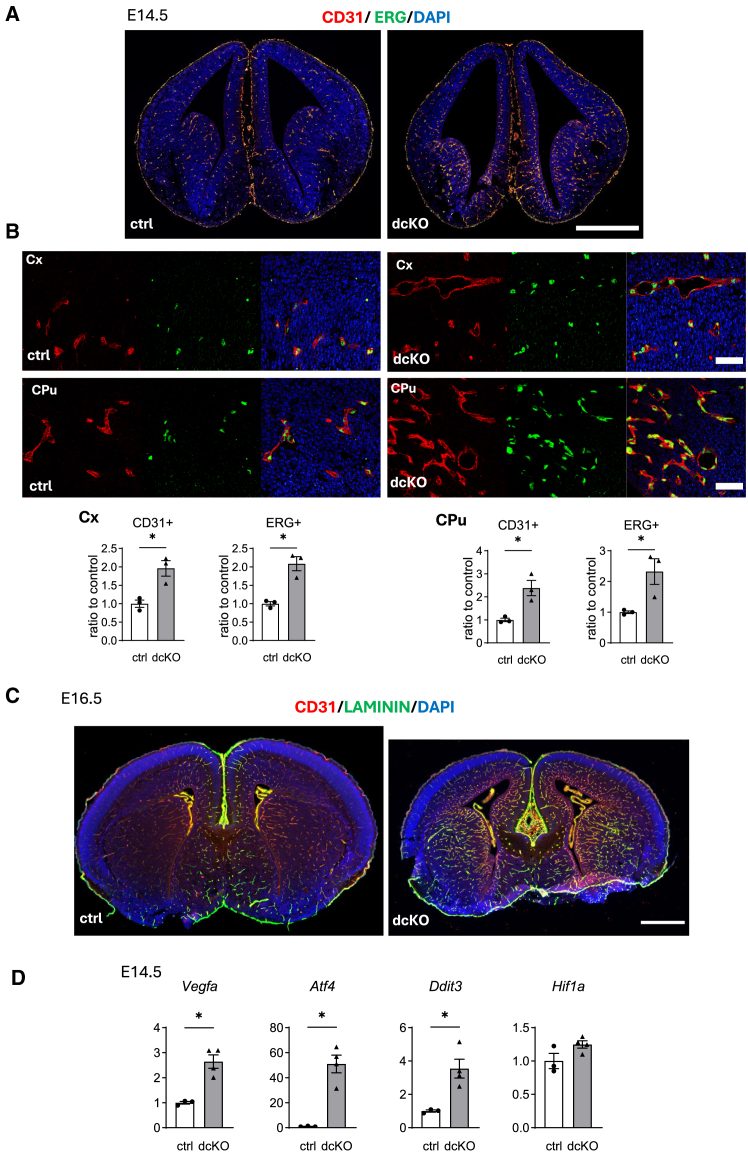
Hypervasculature in dcKO mice (A–C) Brain sections from E14.5 (A and B) and E16.5 (C) embryos were subjected to immunohistochemistry for the indicated molecules. Nuclei were visualized with DAPI (blue). (B) Higher magnification in the cortical cortex (Cx) and caudate putamen (CPu) in (A). The area of CD31 (+) cells and the number of ERG (+) cells were measured using ImageJ, as described in the text (*n* = 3 embryos per group). (D) RT-qPCR. Brain samples from E14.5 brains were subjected to RT-qPCR using specific primers for the indicated genes (*n* = 3–4 per group). Data are represented as mean ± SEM. ∗*p* < 0.05 by Mann-Whitney U test. Scale bars: 500 μm (A and C), 50 μm (B).

Consistent with the findings of hypervasculature in EM, immunohistochemistry for CD31 and ERG, the latter a marker of the endothelial nucleus, revealed increased areas and numbers of CD31(+) and ERG(+) endothelial cells, respectively, in the cerebral cortex (Cx), caudate putamen (CPu), and septum of dcKO mice at E14.5 (Figures 4A and 4B) and P0.5 (data not shown). Immunohistochemical analysis of CD31 and laminin, the latter a marker of the basement membrane, revealed similar vascular phenotypes in dcKO mice at E16.5 (Figure 4C).

To analyze the relationship between the hypervasculature and UPR activation in dcKO mice, RT-qPCR was performed using E14.5 brain samples from both genotypes. The expression of *Vegfa* and *Atf4*, key genes for UPR-associated angiogenesis,^21^ was enhanced in dcKO brains, although that of *Hif1a*, a hypoxia-associated inducer of *Vegfa*, was not different between the two species (Figure 4D). The expression of *Ddit3* (CHOP), a gene critical for UPR-associated cell death, was also enhanced in dcKO brains (Figure 4D).

### Disorganized cortical layers and neuronal tracts and neuroinflammation in dcKO mice

**Figure 5:**
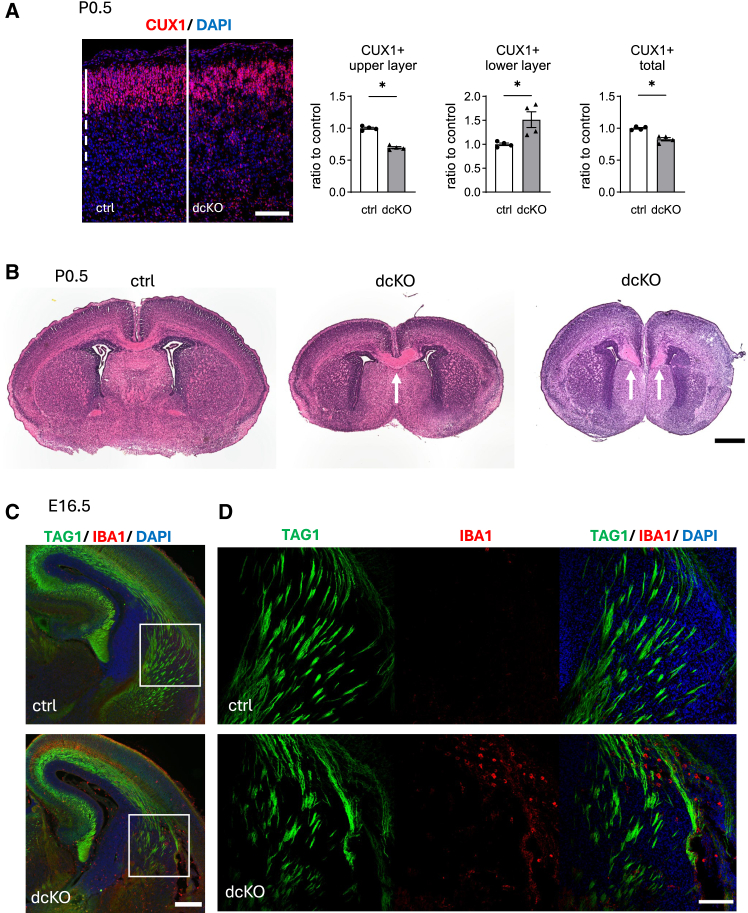
Impaired layer formation and axon projection in dcKO (A and B) Brain sections from P0.5 control or dcKO mice were subjected to immunohistochemistry (A) or Hematoxylin and eosin (H&E) staining (B). (A) CUX1 (a layer II-IV marker)(+) cells were distributed tightly in upper layer in control, but scattered and expressed deeply to lower layer in dcKO mice (*n* = 4 brains per group). Solid line indicates layer II-IV, and dashed line indicates layer V-VI. Data are presented as mean ± SEM. ∗*p* < 0.05 by Mann-Whitney U test. (B) H&E staining showing abnormal corpus callosum (CC) in dcKO brains. Arrows indicate thicken CC (middle) and Probst bundles (right). (C and D) Immunohistochemistry of brain sections from E16.5 control and dcKO embryos for the indicated molecules. Axon direction is parallel in control, but disorganized and surrounded by IBA1(+) macrophages/microglia in dcKO brains (*n* = 4 brains per group). Scale bars: 100 μm (A and D), 500 μm (B), 200 μm (C).

As GRP78-mutant mice have defects in neocortical layer formation^6^^,^^7^ and neuronal tract formation,^7^ the brains of dcKO mice were analyzed at P0.5. Immunohistochemistry for CUX1, a marker of cortical upper layers, revealed that the numbers of CUX1(+) neurons in the total and upper layers (Figure 5A solid line) were reduced in dcKO mice, whereas those in the lower layers (Figure 5A dashed line) were increased in the same mice. These results suggest impaired neuronal migration, in addition to reduced neurogenesis, in dcKO mice. Immunohistochemistry for TBR1, a lower layer marker, revealed that the number of TBR1(+) neurons was slightly but significantly lower in dcKO mice (Figure S4A).

Neuronal tract formation was also analyzed. H&E staining revealed that the corpus callosum (CC), which includes commissural fibers connecting the cerebral cortices on both sides, was enlarged (9 pups/21 pups) or disrupted, leading to the formation of a Probst bundle (4 pups/21 pups) in dcKO mice at P0.5 (Figure 5B). These phenotypes were not observed in control mice (20 pups/20 pups). Consistent with these results, immunohistochemistry revealed disorganization of TAG-1(+) cortical tracts, leading to misdirection or a reduced number of bundles in dcKO mice at P0.5 (Figures 5C, 5D, and S4B). Notably, these neuronal tract phenotypes correlated with the accumulation of IBA1-positive microglia/macrophages (Figure 5D). In contrast to the differences in these cortical tracts, the lateral olfactory tracts, also detected by TAG-1 immunohistochemistry, were observed in a similar manner in both genotypes, although there was greater accumulation of IBA1(+) microglia/macrophages in dcKO brains (Figure S4C).

### Expression profiles of dcKO brains

**Figure 6:**
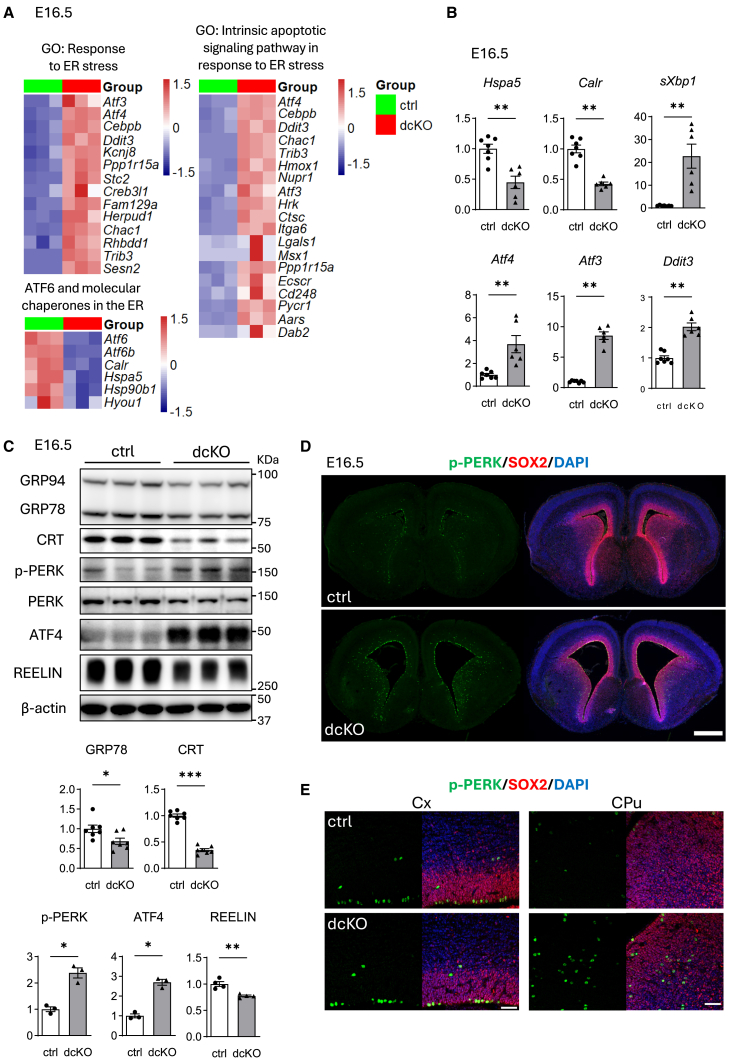
Expression of UPR genes in control and dcKO brains (A) Total RNA isolated from E16.5 control or dcKO cerebral cortex was subjected to RNA-sequencing (*n* = 3 brains per group). Heatmaps show gene expressions in each GO term or in ATF6 branch including molecular chaperones in the ER. Color scale bars indicate *Z* score by row. (B) RT-qPCR. Brains samples from E16.5 control or dcKO cerebral cortex were subjected to RT-qPCR with specific primers for the indicated genes (*n* = 6–7 brains per group). Expressions levels of molecular chaperones in the ER are downregulated, while those of IRE1-and PERK-downstream genes are upregulated in dcKO brains. (C) Western blotting. Protein was extracted from cerebral cortices of E16.5, and subjected to western blotting with indicated antibodies (*n* = 3–7 brains per group). Expression of REELIN was reduced in dcKO brain. (D and E) Immunohistochemistry of brain section from E16.5 control and dcKO embryos for p-PERK and SOX2. p-PERK(+) cells are distributed around ventricular zone (D) and majority of them are co-localized with SOX2(+) cells (E) (*n* = 4 brains per group). Cx, cortex; CPu, caudate putamen. Data are represented as mean ± SEM. ∗*p* < 0.05, ∗∗*p* < 0.01, and ∗∗∗*p* < 0.001 by Mann-Whitney U test. Scale bars: 500 μm (D), 50 μm (E).

To understand gene expression changes upon deletion of ATF6α and ATF6β, RNA sequencing (RNA-seq) was performed using E16.5 brain samples from control and dcKO mice. The expression of 184 genes was upregulated to levels higher than 2-fold and that of 179 genes was downregulated to less than half in dcKO brains (false discovery rate [FDR]-adjusted *p* value of <0.05). Gene ontology (GO) analysis revealed upregulated genes in dcKO mice to be enriched in the terms including “response to endoplasmic reticulum stress” and “intrinsic apoptotic signaling pathway in response to endoplasmic reticulum stress” (Figure 6A). Heatmaps of these genes revealed many PERK-downstream genes, such as *Atf3*, *Atf4*, *Cebpb*, *Ddit3*, *Ppp1r15a*, and *Hmox1* upregulated in dcKO mice (Figure 6A). In contrast, expressions of *Atf6α*, *Atf6b*, and their target genes encoding molecular chaperones in the ER, such as *Hsp5a*, *Hsp90b*, *Calr*, and *Hyou1* were downregulated in the same conditions (Figure 6A). Moreover, expressions of substrates for regulated IRE1-dependent decay of mRNA (RIDD)^22^ were not significantly changed between control and dcKO mice (Figure S5A). Consistently, RT-qPCR revealed the downregulation of molecular chaperones in the ER, as well as the upregulation of IRE1- and PERK-downstream genes, such as *sXbp1*, *Atf4*, *Atf3*, and *Ddit3* in dcKO brains (Figure 6B). Western blot analysis confirmed reduced expression levels of molecular chaperones in the ER and enhanced levels of phosphorylated PERK (p-PERK) and its downstream target, ATF4 (Figure 6C). Furthermore, as described in the previous reports on mutant GRP78,^6^^,^^7^ the expression of REELIN, a large glycoprotein required for the migration and positioning of neurons in the developing brain, was reduced at the protein level (Figure 6C) but not at the transcriptional level (data not shown) in dcKO mice.

To analyze the spatial distribution of activated PERK/ATF4, immunohistochemistry was performed. p-PERK expression was observed predominantly in the SOX2 (+) cells of the regions surrounding the lateral ventricles, including the ventricular zones (Figure 6D). The number of p-PERK (+) cells in the CPu seemed to be higher in the dcKO mice, which was consistent with hypervasculature in the same region (Figure 4C).

In addition to the ER stress-related GO terms aforementioned, those, such as “amino acid metabolic process,” “regulation of angiogenesis,” “response to starvation,” and “inflammatory response” were enriched in dcKO mice. These genes also included PERK-downstream genes, such as *Atf3*, *Atf4*, *Asns*, *Ddit3*, *Ppp1r15a*, and *Hmox1*, (Figure S5A). Consistent with the results in E14.5 brains (Figure 4D), RT-qPCR revealed enhanced level of expression of *Vegfa* in dcKO brains, although no significant difference for that of *Hif1a*, a hypoxia-associated inducer of *Vegfa* (Figure S6A). Furthermore, the expression of *Aif1* (IBA1), a marker of microglia/macrophages, was enhanced in dcKO brains (Figure S6A). Although autophagy is associated with amino acid metabolism and defects of autophagy caused neonatal death,^20^ expressions of autophagy markers or mammalian target of rapamycin (mTOR) substrates were at similar levels between two genotypes at P0.5 (Figure S6B). Consistently, the amounts of ubiquitinated proteins were at similar levels between two genotypes (Figure S6B), and protein aggregates were not observed by immunohistochemistry (data not shown).

**Figure 7:**
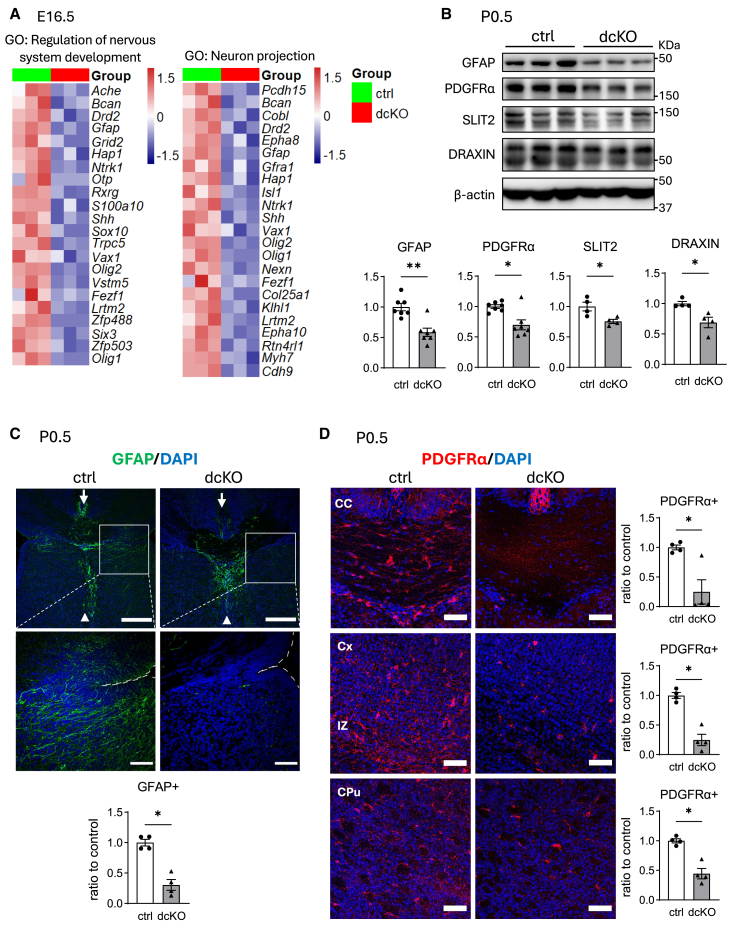
Expression of genes related to brain development in control and dcKO brains (A) Total RNA isolated from E16.5 control or dcKO cerebral cortex was subjected to RNA-sequencing (*n* = 3 brains per group). Heatmaps show gene expressions in each GO term. Color scale bars indicate *Z* score by row. (B) Western blotting. Protein was extracted from cerebral cortex of P0.5 control or dcKO mice, and subjected to western blotting with indicated antibodies (*n* = 4–7 brains per group). (C and D) Immunohistochemistry of brain section from P0.5 control and dcKO mice for GFAP (C) and PDGFRα (D). GFAP(+) Glial wedge (boxed area) are observed in control mice, but not clear in dcKO mice. By contrast, indusium griseum glia (arrow), and midline zipper glia (arrowhead) are observed in both genotypes. GFAP(+) areas in the glial wedge were measured (*n* = 4 brains per group). (D) Oligodendrocyte progenitor cells (PDGFRα(+) cells) are less observed in dcKO mice. The number of PDGFRα(+) cells was counted in each area (*n* = 4 brains per group). CC, corpus callosum; Cx, cortex; IZ, intermediate zone; CPu, caudate putamen. Data are represented as mean ± SEM. ∗*p* < 0.05 and ∗∗*p* < 0.01 by Mann-Whitney U test. Scale bars: 200 μm (C, upper row), 50 μm (C, lower row, D).

In contrast to the upregulated genes, downregulated genes in dcKO mice were enriched in the GO terms, including “behavior,” “regulation of nervous system development,” and “neuron projection development” (Figure S5B). Heatmaps of these genes revealed that they include not only neuronal genes but also glial genes, such as *Gfap* and *S100a10* (astrocytes), and *Shh*, *Sox10*, *Olig1*, and *Olig2* (oligodendrocyte progenitor cells [OPCs]) (Figure 7A). Western blot analysis revealed reduction of GFAP and PDGFRα proteins, the latter also a marker of OPCs, in dcKO mice at P0.5 (Figure 7B).

### Impaired glial development in dcKO brains

In the late embryonic stages, repulsive axon guidance molecules such as SLIT-2^23^ and DRAXIN^24^ are produced in the glial wedge (GW), a midline glia that contributes to CC formation.^25^ Therefore, the expressions of these molecules were analyzed in our model. Western blot analysis revealed that these levels were lower in dcKO brains than in control brains (Figure 7B). As the GW is derived from the radial glia and expresses NESTIN,^26^ its developmental status was compared between the two genotypes. Immunohistochemistry revealed GFAP(+) cells with long processes in the GW of control mice but not in the GW of dcKO mice (Figure 7C). In contrast, glia within the indusium griseum (arrows) and the midline zipper glia (arrowheads), which were other groups of midline glia with different structure and gene expression profiles, were observed in both genotypes (Figure 7C).

During brain development, OPCs are first produced in the ventral forebrain such as the medial ganglion eminence (MGE) and anterior entopeduncular area (AEP), and then migrate to the entire telencephalon including the cerebral cortex. Immunohistochemistry revealed that many PDGFRα(+) OPCs were observed in the white matter such as CC and intermediate region (IZ) and, to a lesser extent, in the gray matter and CPu in the control mice. In contrast, these cells were barely observed in the CC or observed, but to a much lesser extent, in the cortex and CPu of dcKO mice (Figure 7D).

#### Partial rescue of dcKO mice by a chemical chaperone 4-phenylbutyric acid

**Figure 8:**
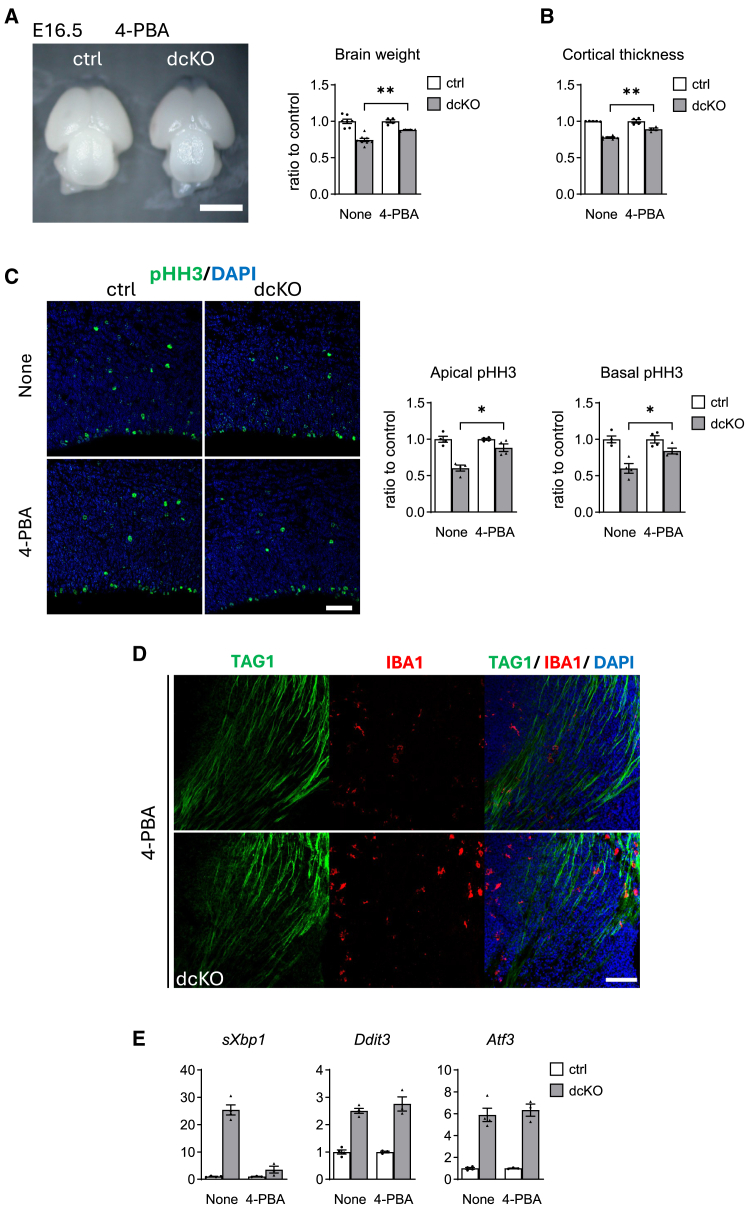
Rescue experiment using 4-PBA. Pregnant mice were administered with 4-PBA from E10.5 to E15.5 (A) Brains from E16.5 embryos (*n* = 4–8 brains per group). (B) Cortical thickness was measured by the thickness of DAPI positive area from immunohistochemistry brain section (*n* = 4–6 brain per group). (C and D) Immunohistochemistry of brain section from E16.5 control and dcKO embryos for the indicated molecules. (C) Note that the number of proliferating cells in dcKO brains increases by 4-PBA both in the apical and basal areas (*n* = 4 brains per group).(D) Axon direction in dcKO brains is also improved to the parallel pattern by 4-PBA, but is still surrounded by IBA1(+) macrophages/microglia (*n* = 4 brains per group). (E) RT-qPCR. Expression of IRE1-downstream gene *(sXbp1)* in dcKO brains decreases by 4-PBA, while that of PERK-downstream genes *(Ddit3* and *Atf3)* does not change (*n* = 3–4 brains per group). Data are represented as mean ± SEM. ∗*p* < 0.05 and ∗∗*p* < 0.01 by Mann-Whitney U test. None: non-treatment group, 4-PBA: treatment group. Scale bars: 2 mm (A), 50 μm (C), 100 μm (D).

As chemical chaperones can alleviate ER stress and rescue pathologies in the CNS, 4-phenylbutyric acid (4-PBA), a commonly used chemical chaperone, was administered from E10.5 to E15.5, as previously described,^27^ followed by evaluation of brains at E16.5. The weights of dcKO brains improved from 74% to 88% of control brains (Figure 8A). Consistent with this result, cortical thickness (Figure 8B), cell proliferation (Figure 8C), and neuronal tract formation (Figure 8D) recovered by 4-PBA. In contrast, cell death (Figure S7A), hypervasculature (Figure S7B), and inflammation (Figure 8D) were not likely recovered. In the gene expression analysis, higher level *sXbp1* expression, but not that of *Ddit3* or *Atf3* expression in dcKO mice, were alleviated by 4-PBA (Figure 8E).

## Discussion

**Figure 9:**
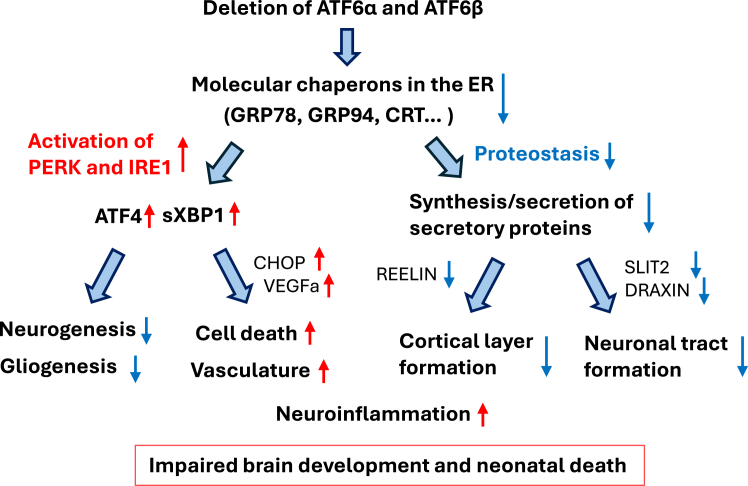
Scheme for the roles of ATF6α and ATF6β in the developing brain Deletion of *Atf6a* and *Atf6b* genes causes reduced levels of expression of molecular chaperones in the ER, leading to hyperactivation of two other UPR branches and impairment of proteostasis. These changes disrupt the microenvironment in the developing brain, resulting in microcephaly and possibly neonatal death.

We have developed *Nes-Cre Atf6α/Atf6b* (dcKO) mice to study the role of ATF6 in the central nervous system. Deletion of both ATF6α and ATF6β in the developing brain caused a variety of phenotypes in neurons, glia, and vessels, as summarized in Figure 9.

Reduced expression levels of molecular chaperones in the ER and increased activation of IRE1 and PERK branches of the UPR were confirmed in dcKO brains using RNA-seq, RT-qPCR, and western blot analysis (Figures 4D and 6A–6C). These results suggested more severe ER stress in the dcKO brains. However, EM analysis did not reveal a clear difference in the structure of the ER, typically enlarged ER in stressed cells, in the two genotypes (Figure S3B). Instead, EM (Figure S3A) and immunohistochemistry (Figures 4A–4C) revealed hypervasculature in the dcKO brains. This hypervasculature likely occurred through PERK/ATF4/VEGFA signals not through HIF1α/VEGFA signals (Figures 4D and S6A). We speculate that, in the ATF6 deleted condition, lower expression levels of molecular chaperones in the ER enhanced the sensitivity of IRE1 and PERK against ER stress by increasing the number of unbound IRE1 and PERK to GRP78, as recently described.^28^ Although the effect of the UPR on vascular development has been reported in tumor cells^29^^,^^30^ and in zebrafish brain,^31^ to the best of our knowledge, this is the first report for the effect of the UPR on vasculature in the developing brain in mammals. Our results also suggest that the activation of the UPR contributes to creating a microenvironment consisting of vasculature, glial cells, immune cells, and substrates, to maintain the survival and the differentiation of neural stem/progenitor cells.

Roles of the UPR in developing brains have been described in different settings, such as neurogenesis,^8^ cortical layer formation,^6^^,^^7^ and axon projection.^7^ In the neurogenesis, deletion of elongator acetyltransferase complex subunit 3 (ELP3) caused ER stress and UPR activation, especially PERK branch.^8^ Indirect neurogenesis, which is conducted by IP in the subventricular zone and plays a critical role in enlarging the brain size in mammals, was specifically reduced in *Elp3* cKO mice. Consistent with this report, the PERK branch was strongly activated (Figures 4D and 6A–6C), and indirect neurogenesis, as determined by the number and the proliferation of IP, was suppressed in our model (Figures 3B–3D). However, there is a discrepancy between the phenotypes of *Elp3* cKO mice and our dcKO mice. In the latter group, the number and proliferation of AP were also reduced (Figures 3A and 3D). Although the cause is not yet clear, one possibility is a higher level of cell death in dcKO mice, which was observed in the ventricular and subventricular zones (Figure 3E). Notably, increased cell death was observed at E14.5, the timing when the structure of the ER was not significantly different between the two genotypes, as described previously.

In the previous reports regarding the GRP78 mutant mice, impaired production/secretion of REELIN was observed in Cajal-Retzius cells.^6^^,^^7^ Consistent with these reports, REELIN expression was reduced at the protein level (Figure 6C), but not at the mRNA levels (data not shown), in our dcKO mice. These results suggest that impaired proteostasis leads to reduced/delayed cortical layer formation through REELIN secretion in dcKO mice (Figure 5A).

Defects in axon projections were also demonstrated in the GRP78 mutant mice, in which neuronal tracts, such as the CC and thalamocortical and corticothalamic tracts, were impaired and disorganized.^7^ Reduced axonal elongation was proposed as a possible mechanism for this defect. Similar defects in axon projections were observed in the dcKO mice (Figures 5B–5D). Moreover, glial development, as determined by the expressions of astrocyte and OPC markers, was impaired in dcKO mice (Figures 7A–7D). The glial wedge, a group of glial cells developing during the embryonic stages from the radial glia, produces axon guidance molecules such as SLIT2 and DRAXIN, and plays a critical role in the formation of the CC. Therefore, axonal projections may be partially associated with the ATF6-dependent glial development.

Taken together with the fact that GRP78 mutants caused similar phenotypes observed in dcKO mice,^6^^,^^7^ it is likely that GRP78 regulates both UPR activation and proteostasis to create a proper microenvironment in the developing brain. However, it remains unclear whether there is a threshold for the expression level of GRP78, or whether there is a critical balance between the expression of GRP78, other chaperones, and the amounts of unfolded proteins. In this regard, it is noteworthy that *Atf6a*^−/−^ mice, in which the expression of GRP78 was reduced to approximately 50–75% of wild-type mice in the brain, developed almost normally in the physiological condition,^10^^,^^32^ while *Atf6β*^−/−^ mice, in which the expression of CRT was specifically reduced to approximately 50% of the wild-type mice in the brain, elicited anxiety-like behavior and hyperactivity.^15^

Administration of 4-PBA improved brain size, neurogenesis, and neuronal tract formation (Figures 8A–8D), while failed to ameliorate cell death, hypervasculature, and inflammation (Figures S7A, S7B and 8D) in dcKO mice. This may be associated with differential effects of 4-PBA on PERK and IRE1 branches (Figure 8E). Considering 4-PBA functioning as a histone deacetylase inhibitor,^33^ it may modulate the stability of sXBP1, as previously described.^34^ Further analysis including the effect of 4-PBA on the neonatal death of dcKO mice will open therapeutic potentials for chemical chaperones to ER stress-associated developmental diseases.

In conclusion, the deletion of ATF6 caused phenotypes in the developing brain, which could be classified as hyperactivation of PERK-/IRE1-associated UPR branches and impaired proteostasis. Our results emphasize critical roles of ATF6 for coordinating the microenvironment required for brain development.

### Limitations of the study

A limitation of the current study is the cause of death in dcKO mice after birth. Although suckling defects have been confirmed as a cause of death, the upstream events, especially those associated with brain development, are unknown. The LOT was observed similarly in both genotypes (Figure S4C), while more IBA1(+) microglia/macrophages were accumulated in the dcKO LOT. Therefore, slight damage to the LOT or other olfactory tracts may affect the suckling behavior in dcKO mice.

Another limitation is the cause of inflammation observed in the dcKO brains. Immunohistochemistry revealed the accumulation of IBA1(+) microglia/macrophages in the neuronal tracts, suggesting an association between neuroinflammation and impaired axonal projection (Figures 5C and 5D). However, 4-PBA administration did not ameliorate accumulation of IBA(+) cells, while neuronal tract formation was improved. Hypervasculature possibly facilitates macrophage infiltration. Further studies are required to confirm this hypothesis.

## Resource availability

### Lead contact

Requests for further information and resources should be directed to and will be fulfilled by the lead contact, Osamu Hori (osamuh3@staff.kanazawa-u.ac.jp).

### Materials availability

The constructs and *Atf6b*^*flox*^ mice generated in this study are available from the lead contact upon request.

### Data and code availability

• The raw data of RNA-sequencing have been deposited at DNA DataBank of Japan (DDBJ) as DRA accession number: PRJDB18856 and are publicly available as of the date of publication.
• Original western blot and microscopy images have been deposited at Mendeley Data and are publicly available at https://doi.org/10.17632/5j2pgxgg73.1 as of the date of publication.
• This study does not report original code.
• Any additional information required to reanalyze the data reported in this study is available from the lead contact upon request.

## Acknowledgments

We are grateful to Dr Gökhan S. Hotamisligil (Harvard School of Public Health) for *Atf6a*^*fl/+*^ mice, Dr. Ryoichiro Kageyama (RIKEN CENTER FOR BRAIN SCIENCE) for *Nes-Cre* mice, and Dr. Hisamichi Naito (Kanazawa University) for valuable discussion. We also thank Dr. Naoki Sakane (Central Pharmaceutical Research Institute, JT Inc.) for the kind gift of anti-ATF6α antibody, clone ATZ-09. This work was supported by a Grant-in Aid for Scientific Research (23K05984 to OH) from the Ministry of Education, Science, Technology, Sports and Culture of Japan, by a grant (20gm1410005s0301) from the Japan Agency for Medical Research and Development (AMED), by the Joint Usage and Joint Research Programs, as well as the Medical Research Center Initiative for High Depth Omics, of the Institute of Advanced Medical Sciences, Tokushima University, and by Kanazawa University CHOZEN project.

## Author contributions

L.D.N. and O.H. conceived and planned the experiments; L.D.N., L.H.N., D.X.D., T.T., K.O., J.Z., and M.M. carried out the experiments; T.H., M.T.-I., H.I., H.K., Y.S., S.Y., S.O., and K.M. supervised the project; O.H. took the lead in writing the manuscript and prepared all figures; all authors provided critical feedback and helped shape the research, analysis, and manuscript.

## Declaration of interests

The authors declare no competing interests.

## Declaration of generative AI and AI-assisted technologies in the writing process

The authors declare no generative AI or AI-assisted tools were used in this study.

## STAR★Methods

### Key resources table

**Table:** 

REAGENT or RESOURCE	SOURCE	IDENTIFIER
**Antibodies**		

Rabbit polyclonal anti-AKT	Enzo Life Sciences	Cat# ADI-KAP-PK004-D; RRID:AB_2038809
Rabbit polyclonal anti-AMPK Alpha	Proteintech	Cat# 10929-2-AP; RRID:AB_2169568
Rabbit monoclonal anti-ATF4 (D4B8)	Cell Signaling Technology	Cat# 11815; RRID:AB_2616025
Mouse monoclonal anti-ATF6α (ATZ-09)	Miyake et al., 2022	N/A
Rat monoclonal anti-ATF6β	BioLegend	Cat# 853202; RRID:AB_2728608
Rat monoclonal anti-CD31 (MEC 13.3)	BD Biosciences	Cat# 550274; RRID:AB_393571
Rabbit monoclonal anti-Calreticulin (D3E6)	Cell Signaling Technology	Cat# 12238; RRID:AB_2688013
Rabbit polyclonal anti-CUX1	Proteintech	Cat# 11733-1-AP; RRID:AB_2086995
Sheep-polyclonal anti-Draxin	R and D Systems	Cat# AF6148; RRID:AB_10571514
Rabbit monoclonal anti-ERG (EPR3864)	Abcam	Cat# ab92513; RRID:AB_2630401
Rabbit polyclonal anti-GFAP	Sigma-Aldrich	Cat# G9269; RRID:AB_477035
Rabbit polyclonal anti-IBA1	FUJIFILM Wako Pure Chemical Corporation	Cat# 019-19741; RRID:AB_839504
Goat polyclonal anti-IBA1	Abcam	Cat# ab5076; RRID:AB_2224402
Mouse monoclonal anti-KDEL	MBL International	Cat# M181-3; RRID:AB_10693914
Rabbit polyclonal anti-LAMININ	Sigma-Aldrich	Cat# L9393; RRID:AB_477163
Rabbit polyclonal anti-LC3	Proteintech	Cat# 14600-1-AP; RRID:AB_2137737
Rabbit Anti-SQSTM1/A170/p62	FUJIFILM Wako Pure Chemical Corporation	Cat# 018-22141; RRID:AB_10658438
Rabbit polyclonal anti-Phospho-Akt (Ser473)	Cell Signaling Technology	Cat# 9271; RRID:AB_329825
Rabbit monoclonal anti-phos-AMPKα (Thr172)	Sigma-Aldrich	Cat# ZRB1213; RRID:AB_3676550
Rabbit polyclonal anti-PDGFRα (C-20)	Santa Cruz Biotechnology	Cat# sc-338; RRID:AB_631064
Rabbit polyclonal anti-PERK (H-300)	Santa Cruz Biotechnology	Cat# sc-13073; RRID:AB_2230863
Rabbit anti-Phospho-S6 Ribosomal protein (Ser236)	Proteintech	Cat# 80206-1-RR; RRID:AB_2918875
Rabbit polyclonal anti-Phospho-Histone H3 (Ser10)	Cell Signaling Technology	Cat# 9701; RRID:AB_331535
Rabbit polyclonal anti-Phospho-PERK (Thr980)	Cell Signaling Technology	Cat# 3191; RRID:AB_3668728
Goat polyclonal anti-REELIN	R and D Systems	Cat# AF3820; RRID:AB_2253745
Rabbit monoclonal anti-S6 Ribosomal Protein (5G10)	Cell Signaling Technology	Cat# 2217; RRID:AB_331355
Rabbit polyclonal anti-SLIT2-Specific	Proteintech	Cat# 20217-1-AP; RRID:AB_10805766
Goat polyclonal anti-SOX2 (Y-17)	Santa Cruz Biotechnology	Cat# sc-17320; RRID:AB_2286684
Goat polyclonal anti-TAG1	R and D Systems	Cat# AF1714; RRID:AB_2245173
Rabbit polyclonal anti-TBR1	Abcam	Cat# ab31940; RRID:AB_2200219
Rabbit polyclonal anti-TBR2	Abcam	Cat# ab23345; RRID:AB_778267
Rabbit polyclonal anti-Ubiquitin	Enzo Life Sciences	Cat# ADI-SPA-200-D; RRID:AB_2039666
Mouse monoclonal anti-β-actin	FUJIFILM Wako Pure Chemical Corporation	Cat# 010-27841; RRID:AB_2858279
Alexa Fluor™ 488 Donkey anti-Rabbit IgG (H+L)	Thermo Fisher Scientific	Cat# A-21206; RRID:AB_2535792
Alexa Fluor™ 488 Donkey anti-Mouse IgG (H+L)	Thermo Fisher Scientific	Cat# A-21202; RRID:AB_141607
Alexa Fluor™ 488 Donkey anti-Goat IgG (H+L)	Thermo Fisher Scientific	Cat# A-11055; RRID:AB_2534102
Cy™3 AffiniPure™ Donkey Anti-Rabbit IgG (H+L)	Jackson ImmunoResearch Labs	Cat# 711-165-152; RRID:AB_2307443
Cy™3 AffiniPure™ Donkey Anti-Mouse IgG (H+L)	Jackson ImmunoResearch Labs	Cat# 715-165-150; RRID:AB_2340813
Cy™3 AffiniPure™ Donkey Anti-Goat IgG (H+L)	Jackson ImmunoResearch Labs	Cat# 705-165-147; RRID:AB_2307351
Cy™3 AffiniPure™ Donkey Anti-Rat IgG (H+L)	Jackson ImmunoResearch Labs	Cat# 712-165-153; RRID:AB_2340667
Mouse anti-rabbit IgG-HRP	Santa Cruz Biotechnology	Cat# sc-2357; RRID:AB_628497
Mouse anti-goat IgG-HRP	Santa Cruz Biotechnology	Cat# sc-2354; RRID:AB_628490
Goat anti-Mouse IgG (H+L) HRP	Thermo Fisher Scientific	Cat# 31431; RRID:AB_10960845

**Biological samples**		

Mouse brain tissue	This study	N/A
RENKA ES cell genomic DNA	Mishina and Sakimura, 2007	N/A

**Chemicals, peptides, and recombinant proteins**		

Bovine serum albumin	FUJIFILM Wako Pure Chemical Corporation	Cat# 015-21274
Immobilon Forte Western HRP substrate	Sigma-Aldrich	Cat# WBLUF0100
Hematoxylin	MUTO PURE CHEMICALS CO., LTD.	Cat# 30002
Eosin	MUTO PURE CHEMICALS CO., LTD.	Cat# 32042
4′,6-diamidino-2-phenylindole	Sigma-Aldrich	Cat# D9542
Epon 812 resin	TAAB Laboratories Equipment Ltd.	Cat# T031
5-ethynyl-2′-deoxyuridine	FUJIFILM Wako Pure Chemical Corporation	Cat# 052-08843
5-Carboxytetramethylrhodamine Azide	MedChemExpress LLC	Cat# HY-151857
Progesterone	Sigma-Aldrich	Cat# P8783-1G
Phenylbutyrate Sodium (4-PBA)	LKT Laboratories, Inc.	Cat# P2815

**Critical commercial assays**		

FASTGene™ RNA Basic Kit	Nippon Genetics Co., Ltd.	Cat# FG-80250
High-Capacity cDNA Reverse Transcription Kit	Thermo Fisher Scientific	Cat# 4374966
THUNDERBIRD® SYBR™ qPCR Mix	TOYOBO Co., Ltd	Cat# QPS-201
ApopTag® Fluorescein *In Situ* Apoptosis Detection Kit	Sigma-Aldrich	Cat# S7110
NEBNext® Ultra™ II Directional RNA Library Prep Kit for Illumina®	New England Biolabs	Cat# E7760L

**Deposited data**		

Raw data for RNA-sequencing	This study	PRJDB18856 (DNA Data Bank of Japan https://ddbj.nig.ac.jp/)
Original data for WB, immunohistochemistry, and photo	This study	https://doi.org/10.17632/5j2pgxgg73.1

**Experimental models: Organisms/strains**		

Mouse: C57BL6/N	Japan SLC, Inc.	N/A
Mouse: B6.Cg-*Atf6*^*tm1Hota*^/J	JAX	RRID:MGI:5699761
Mouse: C57BL6/N Atf6b^f/f^	This study	N/A
Mouse: Tg(Nes-cre)1Kag	Isaka et al., 1999	RRID:MGI:3840982

**Oligonucleotides**		

*sc3AR3*: 5′-AAG TCA GGA GGA GGG TCA CAT CTT AC-3′	This study	N/A
*neoMS02*: 5′-TTC GCA GCG CAT CGC CTT CTA TCG-3′	This study	N/A
*sc5AF3*: 5′-GGG CCA TTT AAA GGC CTT ATA CCG-3′	This study	N/A
*GFP-R10*: 5′-TGA ACT TGT GGC CGT TTA CGT CG-3′	This study	N/A
*F25005*: 5′-AGT GGG CCA GGT GCT TGG CAC-3′	This study	N/A
*R25176*: 5′-TTG ACA CTG TCT CTC AGC AGC CTG-3′	This study	N/A
*F24412*: 5′-CCT GAG AGG AAG AGC ATT GTT CCA G-3′	This study	N/A
*F255279*: 5′-TTA CAC GGG TGT GTC GGG GCA G-3′	This study	N/A
Primers for qPCR, see Table S1	This study	N/A

**Software and algorithms**		

Adobe Photoshop 2024	Adobe	RRID:SCR_014199
GraphPad Prism 10	GraphPad Prism	RRID:SCR_002798
ImageJ 1.50i	ImageJ	RRID:SCR_003070
QuantStudioTM Real-Time PCR Software v1.6.1	Thermo Fisher Scientific	https://www.thermofisher.com/jp/en/home/technical-resources/software-downloads/applied-biosystems-viia-7-real-time-pcr-system.html
Nikon EZ-C1	Nikon	RRID:SCR_014277
R version 4.4.1	R Foundation for Statistical Computing	RRID:SCR_001905
FastQC v0.11.7	Cock et al., 2010	RRID:SCR_014583
Trimmomatic v0.38	Bolger et al., 2014	RRID:SCR_011848
HISAT2 v2.1.0	Kim et al., 2015	RRID:SCR_015530
featureCounts v1.6.3	Liao et al., 2014	RRID:SCR_012919
DESeq2 v1.26.0	Love et al., 2014	RRID:SCR_015687
pheatmap v1.0.12	R-Project	RRID:SCR_016418
Metascape	Metascape	RRID:SCR_016620

**Other**		

Artificial milk for pet	Marukan Group	Cat# MR-146
Plastic Feeding Tube, 22ga x 25mm	Instech Laboratories, Inc.	Cat# FTP-22-25-50

### Experimental model and study participant details

#### Animals

All animal experiments were approved by the Animal Care and Use Committee of Kanazawa University (Approval No. AP-234423). They were conducted in accordance with the Fundamental Guidelines for Proper Conduct of Animal Experiment and Related Activities in Academic Research Institutions under the jurisdiction of the Ministry of Education, Culture, Sports, Science and Technology, as well as in compliance with the ARRIVE guidelines. Mice were housed in a temperature and humidity-controlled environment under a 12-hours light-dark cycle at the Institute for Experimental Animals, Advanced Research Center, Kanazawa University.

*Atf6a*^*fl/+*^ mice^18^ and *Nes-Cre* mice^35^ were developed, as previously described, and provided by the Jackson laboratory and by Institute of Resource Development and Analysis, Center for Animal Resources and Development, Kumamoto University, respectively. They were backcrossed in C57BL/6N genetic background for more than 8 times. *Atf6b*^*fl/+*^ mice were generated in TransGenic Inc., as described below and in Figure S1. C57BL/6N mice were used as wild-type mice. *Atf6a*^*fl/fl*^*Atf6b*^*fl/fl*^ mice were obtained by crossing *Atf6a*^*fl/fl*^ and *Atf6b*^*fl/fl*^ mice. To obtain *Nes-Cre Atf6a*^*fl/fl*^*Atf6b*^*fl/fl*^ (*Atf6α/Atf6b* double conditional knockout (dcKO)) mice, *Atf6a*^*fl/fl*^*Atf6b*^*fl/f*^ female mice were crossed with either *Nes-Cre Atf6a*^*fl/+*^*Atf6b*^*fl/f*^ or *Nes-Cre Atf6a*^*fl/fl*^*Atf6b*^*fl/+*^ male mice. *Atf6a*^*fl/fl*^*Atf6b*^*fl/fl*^ mice were used as control mice in most experiments, but in some cases, *Atf6a*^*fl/fl*^*Atf6b*^*fl/+*^ mice were also used as control mice due to the limited numbers of littermates.

Mice were mated as above at 6:00 pm and checked for vaginal plug at 9:00 am next morning. Gestational day zero (E0.5) was identified as the morning on which the plug was found. All embryos and pups were collected regardless of gender.

### Method details

#### Generation of *Atf6b*^*fl/+*^ mice

To generate *Atf6b* gene “floxed” mice (*Atf6b*^*fl/+*^ mice) for conditional knockout, two loxP sequences were inserted into mouse *Atf6b* locus by homologous recombination. To construct the targeting vector, a 5.0 kb mouse genomic fragment containing the sequence from upstream region of exon 1 to intron 9, a 0.9 kb fragment containing the sequence from intron 9 to intron 11, and 3.8 kb fragment containing the sequence from intron 11 to 3’ region of *Atf6b* gene were amplified by PCR from RENKA ES cell genomic DNA.^36^ The 0.9 kb fragment was added with loxP sequence in PCR primer on the 3′ end. These 5.0 kb, 0.9 kb and 3.8 kb genomic fragments were cloned into a plasmid vector including MC1_DTA cassette (MC1 promoter driven diphtheria toxin A gene) as negative selection marker. Subsequently, “knockout first cassette” which containing frt, En2 splicing acceptor, internal ribosomal entry site (IRES), EGFP, SV40 polyA, loxP, human beta actin promoter, neomycin resistant gene, phosphoglycerate kinase poly A, frt and loxP, was inserted at 5′ end of the 0.9 kb fragment. Resulting targeting vector contains 5.0 kb 5′ homologous arm, frt flanked knockout first cassette including neomycin resistant gene, loxP site, 0.9 kb floxed genomic region containing exon 10 and 11 of *Atf6b* gene, loxP site, 3.8 kb 3′ homologous arm and MC1_DT cassette.

This targeting vector was linearized and introduced into RENKA ES cells (C57BL/6N) by electroporation. After selection using Geneticin, the resistant clones were isolated, and their DNA samples were screened for homologous recombinant by PCR using following primer sets: *sc3AR3* and *neoMS02*. PCR positive ES clones were expanded, and isolated DNA samples were further analyzed by PCR amplification using following primer sets: *sc5AF3* and *GFP-R10* for 5′ amplification, *sc3AR3* and *neoMS02* for 3′ amplification, and *F25005* and *R25176* for amplification of loxP region. Homologous recombination of these clones was also confirmed by genomic Southern hybridization probed with neomycin resistant gene.

Homologous recombinant ES cell clones were aggregated with ICR 8 cell stage embryos to generate chimeric mice. Germline transmitted F1 heterozygous mice were obtained by crossing between chimeric mice with high contribution of the RENKA background and C57BL/6N mice. Targeted allele was identified by PCR with primer set, *sc3AR3* and *neoMS02*.

In order to remove the knockout first cassette to produce *Atf6b*^*fl/+*^ mice, F1 heterozygous mice were crossed with *ROSA26 CAG-Flp* knock-in mice which express Flp in germ cells. Floxed allele without knockout first cassette was identified with following PCR primer set, *F24412* and *F255279*.

#### RT-qPCR

Total RNA was extracted from cortices of embryonic and neonatal mouse brains using FASTGene™ RNA Basic Kit (Nippon Genetics). Reverse transcription reactions were performed using High-Capacity cDNA Reverse Transcription Kit (Thermo Fisher Scientific). Individual cDNAs were amplified with THUNDERBIRD™ SYBR qPCR Mix (TOYOBO) using specific primers whose sequences are listed in Table S1. The comparative Ct method was used for data analyses with QuantStudio™ Real-Time PCR Software v1.6.1 (Thermo Fisher Scientific). Values for each gene were normalized against the *Gapdh* expression level.

#### Western blotting

Protein samples from cortices of the indicated mouse brains were solubilized in RIPA buffer, which contained 10 mM Tris (pH 7.6), 1 mM EDTA, 150 mM NaCl, 1% NP-40, 0.1% SDS, 0.2% sodium deoxycholate, 1 mM PMSF, 1μg/ml aprotinin, 10 mM NaF, and 1 mM Na_3_VO_4_. After electrophoresis and transferring, membranes were incubated with 3% bovine serum albumin for 1 h and then with the primary antibodies for overnight at 4°C. The primary antibodies used are listed in Key resources table except anti-ATF6α antibody, clone ATZ-09, which was produced, as previously described.^37^ Sites of primary antibody binding were determined using an enhanced chemiluminescence system (GE Healthcare). Horseradish peroxidase (HRP)-conjugated secondary antibodies (Santa Cruz Biotechnology) were used to detect primary antibodies.

#### Histology and immunohistochemistry

Brains were dissected from E16.5 and P0.5 mice after transcardiac perfusion with phosphate-buffered saline (PBS) followed by 4% paraformaldehyde prepared in 0.1 M phosphate buffer (pH 7.4), and from E14.5 mice without perfusion. Coronal sections with 10 or 20 μm thickness were cut on a cryostat (Leica Biosystems). Sections were processed for H&E staining (MUTO PURE CHEMICALS), TUNEL assay (ApopTag® Fluorescein *In Situ* Apoptosis Detection Kit, Sigma-Aldrich) or immunohistochemistry with antibodies listed in Key resources table. Secondary antibodies were used to visualize immunolabeling including anti-rabbit, anti-mouse, and anti-goat Alexa Fluor 488-conjugated (Thermo Fisher Scientific) and Cy3-conjugated (Jackson ImmunoResearch Laboratories, Inc., West Grove, PA, USA). Nuclei were visualized with 4′,6-diamidino-2-phenylindole (DAPI, Sigma-Aldrich). Imaging was performed using laser scanning confocal microscope (Eclipse TE200U; Nikon) and Nikon EZ-C1 software or using light and fluorescence microscope (BZ-X700; Keyence).

#### 5-ethynyl-2′-deoxyuridine (EdU) incorporation

EdU incorporation was performed, as described previously.^38^ In brief, pregnant mice were intraperitoneally injected with 50 mg/kg EdU (FUJIFILM Wako Pure Chemical) on E14.5, and embryonic brains were dissected and underwent tissue processing after 1 h. EdU was visualized by incubating in the EdU staining buffer containing 0.5 μM 5-Carboxytetramethylrhodamine Azide (5-TAMRA-Azide) (MedChemExpress), 0.5 mM CuSO4, and 50 mM ascorbate in PBS.

#### 4-phenylbutyrate acid (4-PBA) administration

Sodium 4-phenylbutyrate (LKT laboratories), dissolved in saline, was intraperitoneally administered to pregnant mice at 100 mg/kg body weight, once a day from E10.5 to E15.5, as previously described.^27^ Brain samples were prepared at E16.5, as described above.

#### RNA-sequencing

The preparation of a cDNA library and sequencing was conducted by Rhelixa Co., Ltd. Messenger RNAs purified from cortices of control and *Atf6α/Atf6b* dcKO embryos (n=3 per group) were used to prepare cDNA libraries with the use of a NEBNext Ultra II Directional RNA Library Prep Kit for Illumina (New England Biolabs). Each library was sequenced with the use of a NovaSeq 6000 system (Illumina). The quality of the raw sequencing data was checked with FastQC (version 0.11.7),^39^ and trimming of the adapter sequences was performed with Trimmomatic (version 0.38).^40^ The total amount of each mRNA was calculated with the use of a series of programs including HISAT2 (version 2.1.0),^41^ featureCounts (version 1.6.3),^42^ and DESeq2 (version 1.26.0).^43^ RNA-seq reads were mapped against the mouse (mm10) genome. Differentially expressed genes (DEGs) were detected with the thresholds of |log2FC (Fold Change)| > 1 and adjusted p value < 0.05. Gene ontology analysis was performed using Metascape web tools for DEGs,^44^ and Heatmaps were created from Z-scores of the normalized counts using the pheatmap package (version 1.0.12) in R (version 4.4.1, R Foundation for Statistical Computing). The raw reads are available in the DNA Data Bank of Japan (DDBJ) with DDBJ Sequence Read Archive (DRA) accession number, PRJDB18856.

#### Suckling test

Suckling test was performed as described previously.^19^ In brief, an anesthetized lactating female mouse was placed supine, and each pup was placed 1cm from the female’s nipple with vertical support for 120 s after starvation for 3 h. In some cases, pup was placed directly on the nipple.

#### Analysis of neonatal lethality of mice

Neonatal lethality was analyzed as described previously.^20^ In brief, pregnant mice were subcutaneously injected with 1 mg progesterone (Sigma-Aldrich) on E17.5 and E18.5 to delay the delivery. Newborn pups were delivered by cesarean section on E19.5 and then placed in a humidified chamber with temperature set at 30°C. Pups were monitored every one hour to determine the time of death. In some cases, pups were administered with artificial milk (Marukan Group) with feeding needles (Instech Laboratories, Inc.) every 4-6 hours.

### Quantification and statistical analysis

Quantification in western blot, histology and immunohistochemistry was performed using ImageJ software 1.50i. Mann-Whitney U test or Mantel-Cox test was used for statistical analyses with GraphPad Prism software 10.0. A p-value less than 0.05 was considered statistically significant.
