# Supplementary material for: Impact of ATF6 deletion on the embryonic brain development
Source: iScience. 2025 May 2;28(6):112569. doi: 10.1016/j.isci.2025.112569 (PMC12143657; doi:10.1016/j.isci.2025.112569)
Supplement: Document S1. Figures S1–S9 and Tables S1 [file mmc1.pdf]

iScience, Volume *28*

Supplemental information

Impact of ATF6 deletion

on the embryonic brain development

Loc Dinh Nguyen, Ly Huong Nguyen, Dat Xuan Dao, Tsuyoshi Hattori, Mika Takarada-Iemata, Hiroshi Ishii, Takashi Tamatani, Hiroshi Kawasaki, Yohei Shinmyo, Kenta Onoue, Shigenobu Yonemura, Jun Zhang, Masato Miyake, Seiichi Oyadomari, Kazutoshi Mori, and Osamu Hori

Supplementary information

Table S1 The sequences of primers for RT-qPCR in this study. Related to STAR Methods.

Gene	Forward primer	Reverse primer
Atf6a	ACAGCTACCTAACCATGTG	AGCGATATCCGAACCCATAC
Atf6b	AACAGGAAGGTTGTCTGCATCAT	CGTCTCATCCGAGGAGACATG
Atf3	GAGGATTTTGCTAACCTGACACC	TTGACGGTAACTGACTCCAGC
Atf4	GGACAGATTGGATGTTGGAGA	TAAAGGAATGCTCTGGAGTGG
Atf5	GGCTGGCTCGTAGACTATGG	ACCCGCTCAGTCATCCAATC
Calr	ATAAAGGGCTGCAGACAAGC	CCACAGTCGATATTCTGCTC
Ddit3(CHOP)	AAGTGGCACAGCTAGCTGAA	GCCCACTGTTCATGCTTGGT
Hif1a	ACCTTCATCGGAAACTCCAAAG	CTGTTAGGCTGGGAAAAGTTAGG
Hspa5(GRP78)	ATGGTATTCTCCGAGTGACA	GCTTTCCAGCCATTCAATCT
Aif1(IBA1)	CAGCAATGATGAGGATCTGC	CCAAGTTTCTCCAGCATTCG
Ern1(IRE1a)	TGTGGTCAAGATGGACTGGC	TCGGAGGAGGTCTCTCACAG
Pdgfra	TCCATGCTAGACTCAGAAGTCA	TCCCCGGTGGACACAATTTTTC
Eif2ak3(PERK)	CACGCAGATCACAGTCAGGT	GTGGGGCTGAGGATGGAAAA
Reelin	CCACTCGCCACCAATTCTCC	CAGATCCCTCGTCCTGAGCA
sXbp1	GGTCTGCTGAGTCCGCAGCAGG	CTCTGGGGAAGGACATTTGA
Vegfa	TTACTGCTGTACCTCCAC	ACAGGACGGCTTGAAGATG
Gapdh	ACCCAGAAGACTGTGGATGG	CACATTGGGGGTAGGAACAC

Supplementary Figure Titles and Figure Legends

Figure S1. Generation of *Atf6b*$^{fl/+}$ mice. Related to Figure 1.

(A) Strategy to construct *Atf6b*$^{fl/+}$ mice by homologous recombination. (B)(C) PCR (B) and Southern Blot analysis (C) to confirm the targeted allele. PC: positive control, ddw: double distilled water. (D) PCR to confirm Flp-mediated deletion of neomycin resistant gene (neo) cassette.

Figure S2. Characterization of dcKO neonatal mice. Related to Figure 2. (A) Genotype distribution of living pups from *Nes-Cre Atf6a*$^{fl/fl}$*Atf6b*$^{fl/+}$ (male) and *Atf6a*$^{fl/fl}$*Atf6b*$^{fl/fl}$ (female) parents. (B) Opened abdomen of control and dcKO mice from the same littermate sacrificed at P0.5. Arrows indicate stomach, and asterisks indicate inside stomach with or without milk. (C) Hearts and lungs dissected from mice in (B) indicate similar size, color, and expansion between the two genotypes. (D) H&E staining of lungs from P0.5 control and dcKO mice indicates similar patterns of staining and extension of alveoli in the both genotypes. Scale bar: 200μm.

Figure S3. Electron microscopic analysis. Related to Figure 4. Brain sections including cerebral cortices from E14.5 control and dcKO embryos were subjected to EM analysis. Wide view (low magnification) analysis revealed more vasculature (A, upper row) with similar luminal structure (A, lower row) in dcKO brains. Arrows indicate vasculature. Typical images from 4 brains in each group are shown. Scale bars: 100μm (A), 200μm (B).

Figure S4. Impaired layer formation and axon projection in dcKO mice. Related to Figure 5. Brain sections from P0.5 mice were subjected to immunohistochemistry for the indicated molecules. (A) The number of TBR1 (layer V-VI marker)(+) cells was slightly, but significantly, lower in dcKO brain (n=4 mice per group). Data are shown as mean±SEM. *$p<0.05$ by Mann-Whitney U test. (B) TAG1(+) neuronal tracts are disorganized and IBA1(+) macrophages/microglia are accumulated in dcKO brains (n=4 brains per group). (C) TAG1(+) lateral olfactory tract (LOT: arrows) was observed in a similar manner between two genotypes. Arrowheads: IBA(+) microglia. Scale bars: 100μm (A), 50μm (B), 200μm (C).

Figure S5. GO analysis of RNA-sequencing from E16.5 control and dcKO brain. Related to Figure 6 and 7. Raw data was filtered to collect only genes with adjusted p-value<0.05 and fold-change >2, and GO enrichment analysis was performed to classify genes by GO terms for both upregulated (A) and downregulated genes (B). In (A), Heatmaps show the different expression of genes in the indicated GO terms. Color scale bars indicate z-score by row.

Figure S6. Expression of genes related to vascular formation (A) and autophagy (B) in control and dcKO brain. Related to Figure 6 and 7. (A) Total RNA from E16.5 control or dcKO cerebral cortex was subjected to RT-qPCR with specific primers for indicated genes (n=6-7 brains per group). Data are shown as mean±SEM. **$p<0.01$, ***$p<0.001$ by Mann-Whitney U test. (B) Western blotting. Protein was extracted from cerebral cortex of P0.5 mice, and subjected to western blotting with indicated antibodies (n=3-4 brains per group).

Figure S7. Rescue experiment using 4-PBA. Related to Figure 8. Pregnant mice were treated with 4-PBA as described and sacrificed on E16.5. Brain sections from control or dcKO mice were subjected to TUNEL staining (A) or immunohistochemistry (B) with indicated antibodies. (A) Number of cell death in dcKO was not reduced after 4-PBA treatment (n=3-4 brains per group). Data are shown as mean±SEM. (B) Hypervasculature is still observed in dcKO after 4-PBA treatment. None: mice without 4-PBA administration, 4-PBA: mice with 4-PBA administration. Scale bars: 500μm.

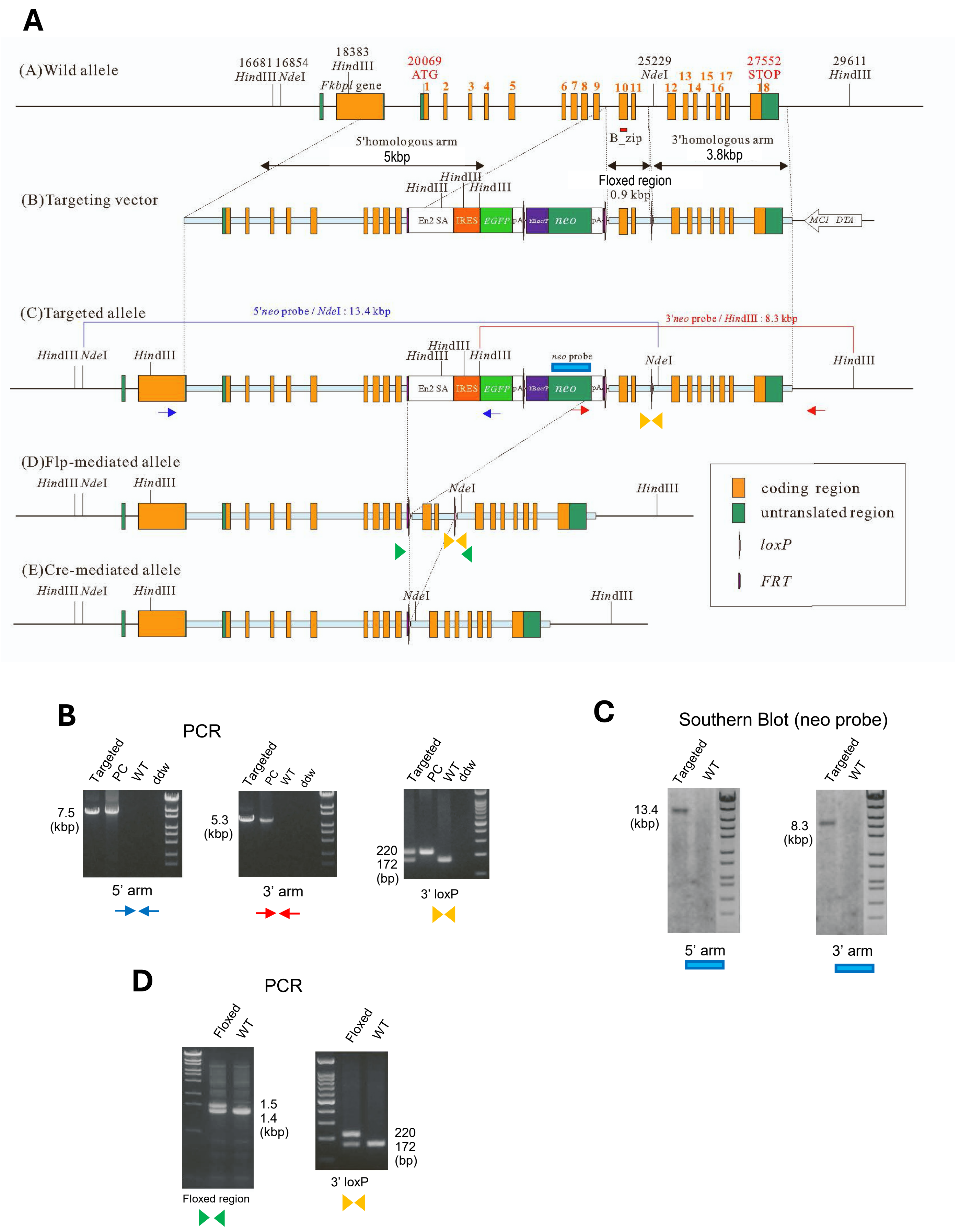
A
(A)Wild allele
16681 16854
HindIII NdeI
18383
HindIII
Fkbpl gene
20069
ATG
25229
NdeI
27552
STOP
29611
HindIII
B_zip
5'homologous arm
5kbp
3'homologous arm
3.8kbp
Floxed region
0.9 kbp
(B)Targeting vector
En2 SA
IRES
EGFP
neo
MC1 DTA
(C)Targeted allele
5'neo probe / NdeI : 13.4 kbp
3'neo probe / HindIII : 8.3 kbp
neo probe
(D)Flp-mediated allele
(E)Cre-mediated allele
coding region
untranslated region
loxP
FRT
B
PCR
Targeted
PC
WT
ddw
7.5
(kbp)
5' arm
5.3
(kbp)
3' arm
220
172
(bp)
3' loxP
C
Southern Blot (neo probe)
13.4
(kbp)
8.3
(kbp)
D
Floxed
1.5
1.4
Floxed region

Figure.S1

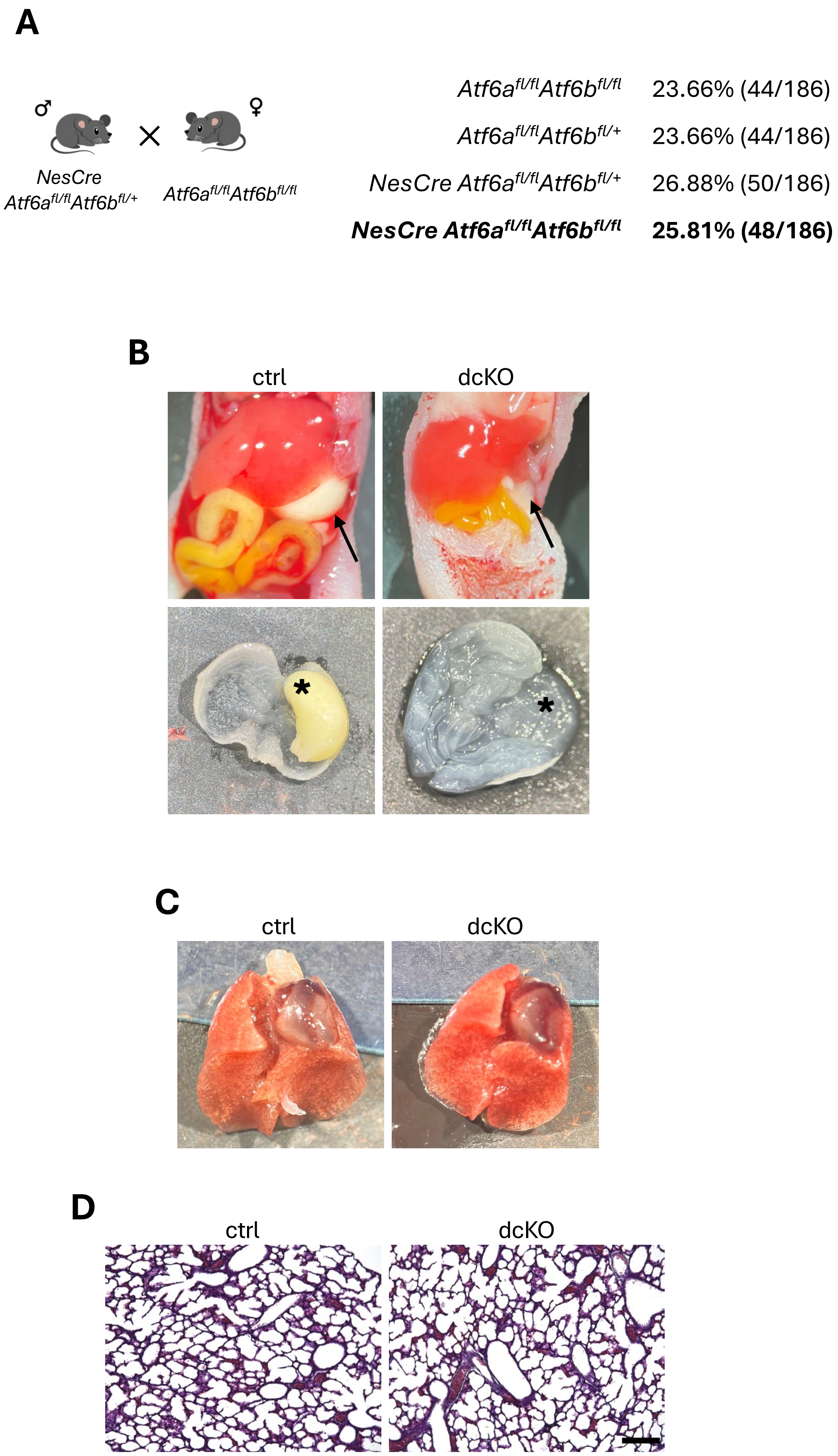
A
♂
♀
×
NesCre
$Atf6a^{fl/fl}Atf6b^{fl/+}$
$Atf6a^{fl/fl}Atf6b^{fl/fl}$
$Atf6a^{fl/fl}Atf6b^{fl/fl}$ 23.66% (44/186)
$Atf6a^{fl/fl}Atf6b^{fl/+}$ 23.66% (44/186)
NesCre $Atf6a^{fl/fl}Atf6b^{fl/+}$ 26.88% (50/186)
NesCre $Atf6a^{fl/fl}Atf6b^{fl/fl}$ 25.81% (48/186)
B
ctrl
dcKO
C
ctrl
dcKO
D
ctrl
dcKO

Figure.S2

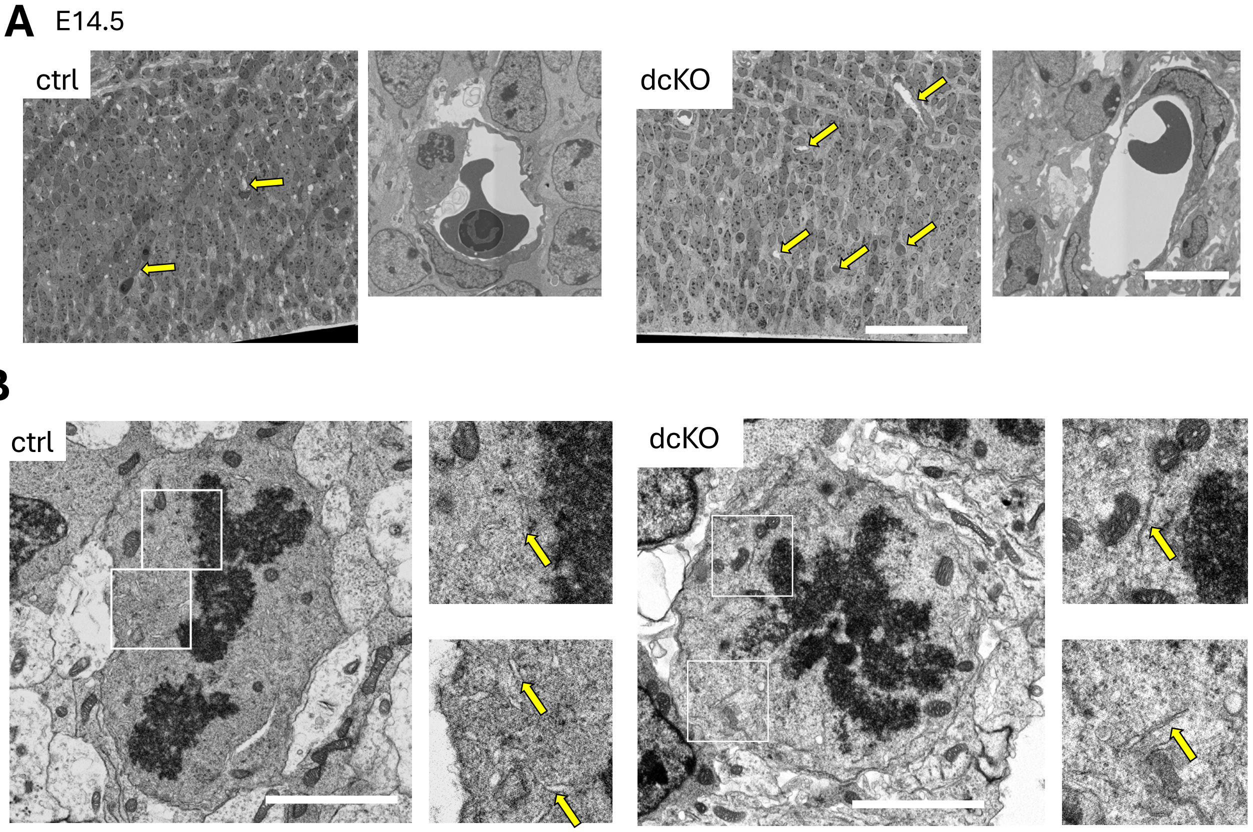

Figure.S3

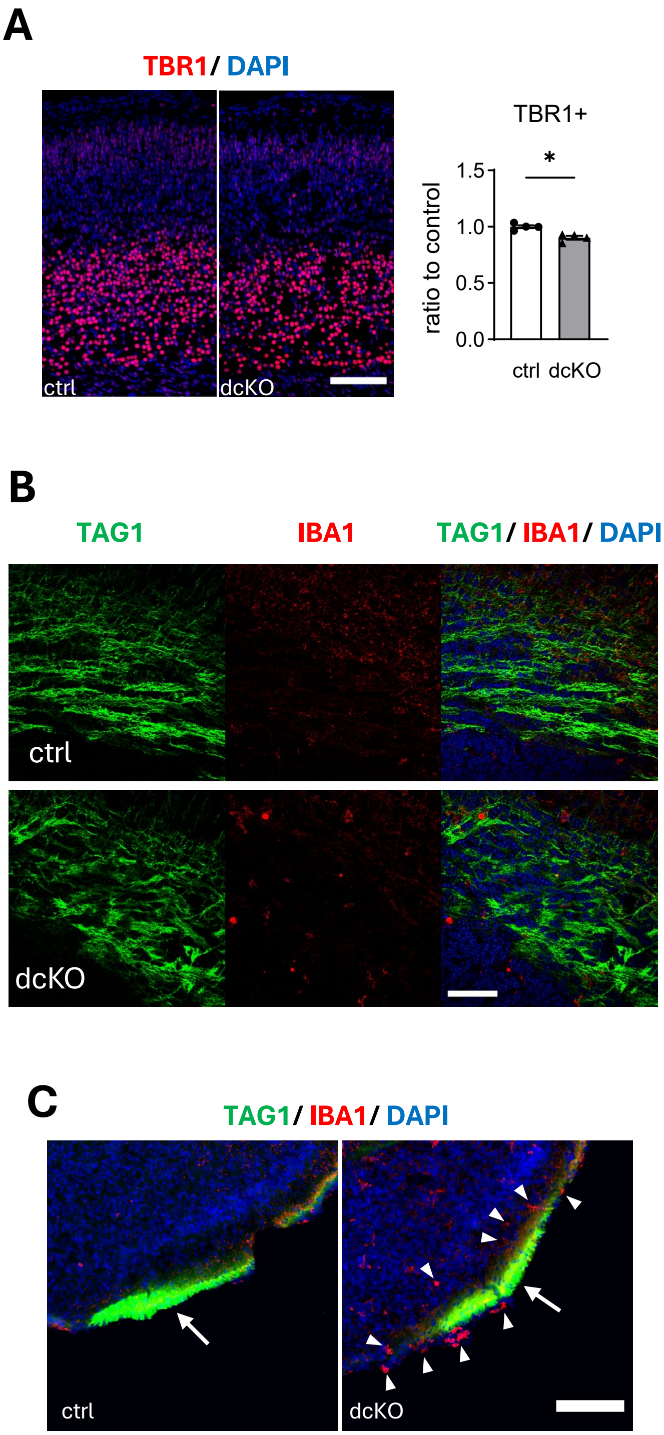
A
TBR1/ DAPI
ctrl
dcKO
TBR1+
*
1.5
1.0
0.5
0.0
ratio to control
ctrl dcKO
B
TAG1
IBA1
TAG1/ IBA1/ DAPI
ctrl
dcKO
C
TAG1/ IBA1/ DAPI
ctrl
dcKO

Figure.S4

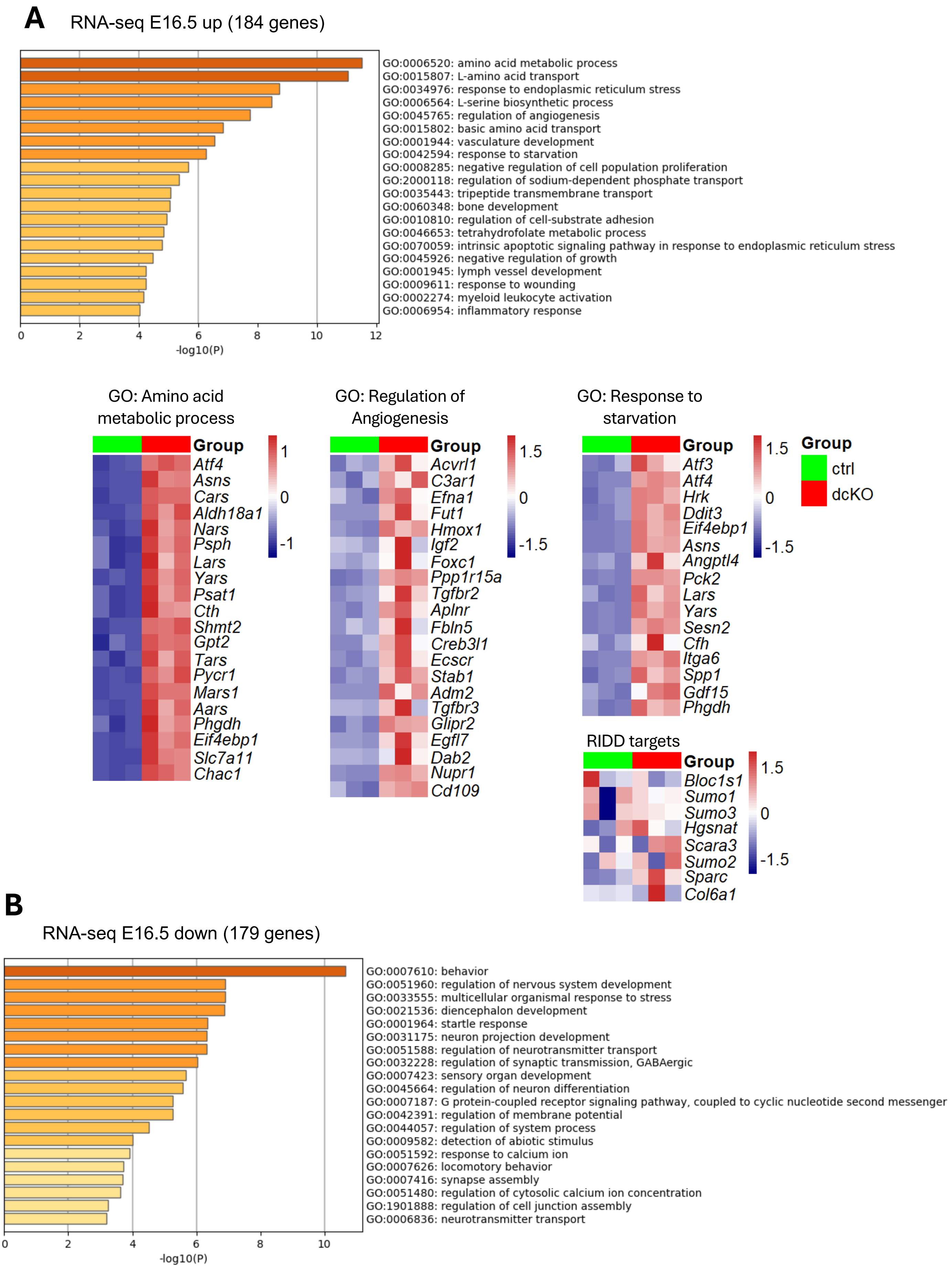
A
RNA-seq E16.5 up (184 genes)
GO:0006520: amino acid metabolic process
GO:0015807: L-amino acid transport
GO:0034976: response to endoplasmic reticulum stress
GO:0006564: L-serine biosynthetic process
GO:0045765: regulation of angiogenesis
GO:0015802: basic amino acid transport
GO:0001944: vasculature development
GO:0042594: response to starvation
GO:0008285: negative regulation of cell population proliferation
GO:2000118: regulation of sodium-dependent phosphate transport
GO:0035443: tripeptide transmembrane transport
GO:0060348: bone development
GO:0010810: regulation of cell-substrate adhesion
GO:0046653: tetrahydrofolate metabolic process
GO:0070059: intrinsic apoptotic signaling pathway in response to endoplasmic reticulum stress
GO:0045926: negative regulation of growth
GO:0001945: lymph vessel development
GO:0009611: response to wounding
GO:0002274: myeloid leukocyte activation
GO:0006954: inflammatory response
0
2
4
6
8
10
12
-log10(P)
GO: Amino acid metabolic process
Group
Atf4
Asns
Cars
Aldh18a1
Nars
Psph
Lars
Yars
Psat1
Cth
Shmt2
Gpt2
Tars
Pycr1
Mars1
Aars
Phgdh
Eif4ebp1
Slc7a11
Chac1
1
0
-1
GO: Regulation of Angiogenesis
Group
Acvrl1
C3ar1
Efna1
Fut1
Hmox1
Igf2
Foxc1
Ppp1r15a
Tgfbr2
Aplnr
Fbln5
Creb3l1
Ecscr
Stab1
Adm2
Tgfbr3
Glipr2
Egfl7
Dab2
Nupr1
Cd109
1.5
0
-1.5
GO: Response to starvation
Group
Atf3
Atf4
Hrk
Ddit3
Eif4ebp1
Asns
Angptl4
Pck2
Lars
Yars
Sesn2
Cfh
Itga6
Spp1
Gdf15
Phgdh
1.5
0
-1.5
Group
ctrl
dcKO
RIDD targets
Group
Bloc1s1
Sumo1
Sumo3
Hgsnat
Scara3
Sumo2
Sparc
Col6a1
1.5
0
-1.5
B
RNA-seq E16.5 down (179 genes)
GO:0007610: behavior
GO:0051960: regulation of nervous system development
GO:0033555: multicellular organismal response to stress
GO:0021536: diencephalon development
GO:0001964: startle response
GO:0031175: neuron projection development
GO:0051588: regulation of neurotransmitter transport
GO:0032228: regulation of synaptic transmission, GABAergic
GO:0007423: sensory organ development
GO:0045664: regulation of neuron differentiation
GO:0007187: G protein-coupled receptor signaling pathway, coupled to cyclic nucleotide second messenger
GO:0042391: regulation of membrane potential
GO:0044057: regulation of system process
GO:0009582: detection of abiotic stimulus
GO:0051592: response to calcium ion
GO:0007626: locomotory behavior
GO:0007416: synapse assembly
GO:0051480: regulation of cytosolic calcium ion concentration
GO:1901888: regulation of cell junction assembly
GO:0006836: neurotransmitter transport
0
2
4
6
8
10
-log10(P)

Figure.S5

A

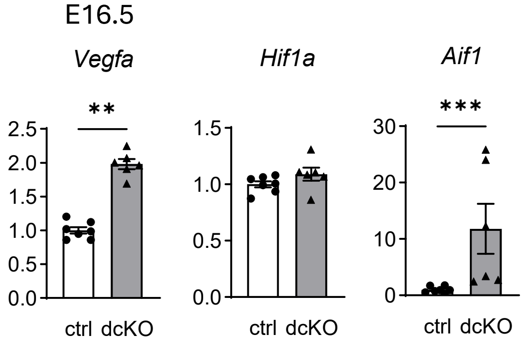

E16.5
Vegfa
Hif1a
Aif1
**
***
2.5
2.0
1.5
1.0
0.5
0.0
1.5
1.0
0.5
0.0
30
20
10
0
ctrl dcKO
ctrl dcKO
ctrl dcKO

B

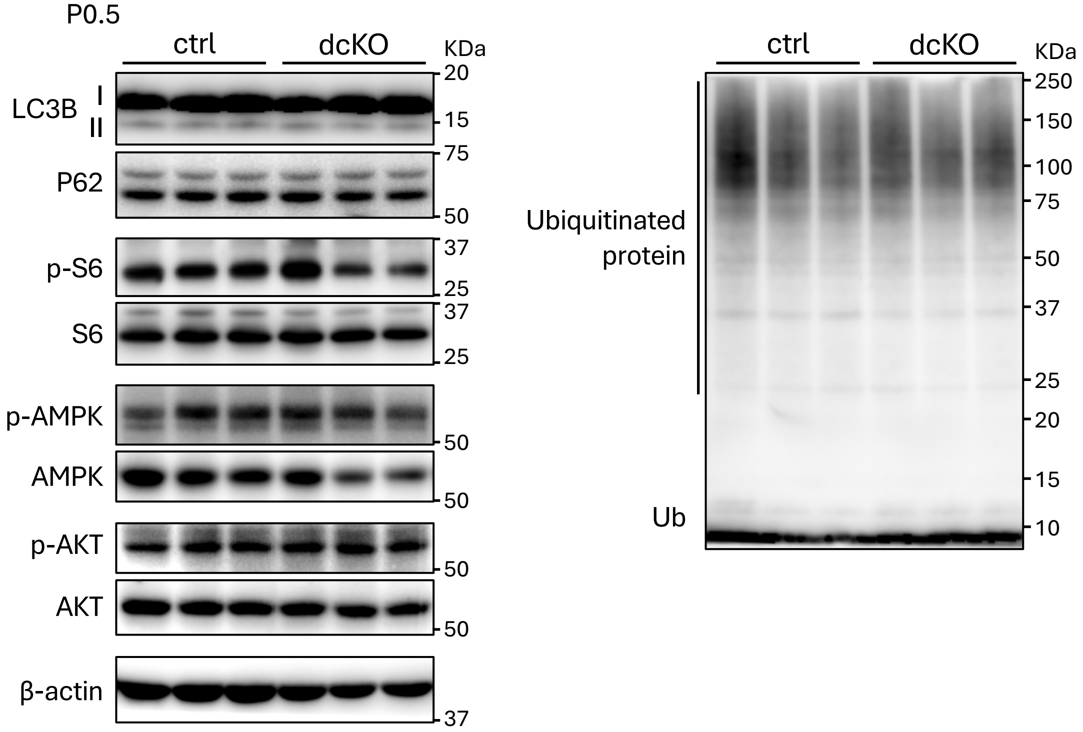

P0.5
ctrl
dcKO
KDa
LC3B
I
II
P62
p-S6
S6
p-AMPK
AMPK
p-AKT
AKT
β-actin
20
15
75
50
37
25
37
25
50
50
50
50
37
ctrl
dcKO
KDa
Ubiquitinated protein
Ub
250
150
100
75
50
37
25
20
15
10

Figure.S6

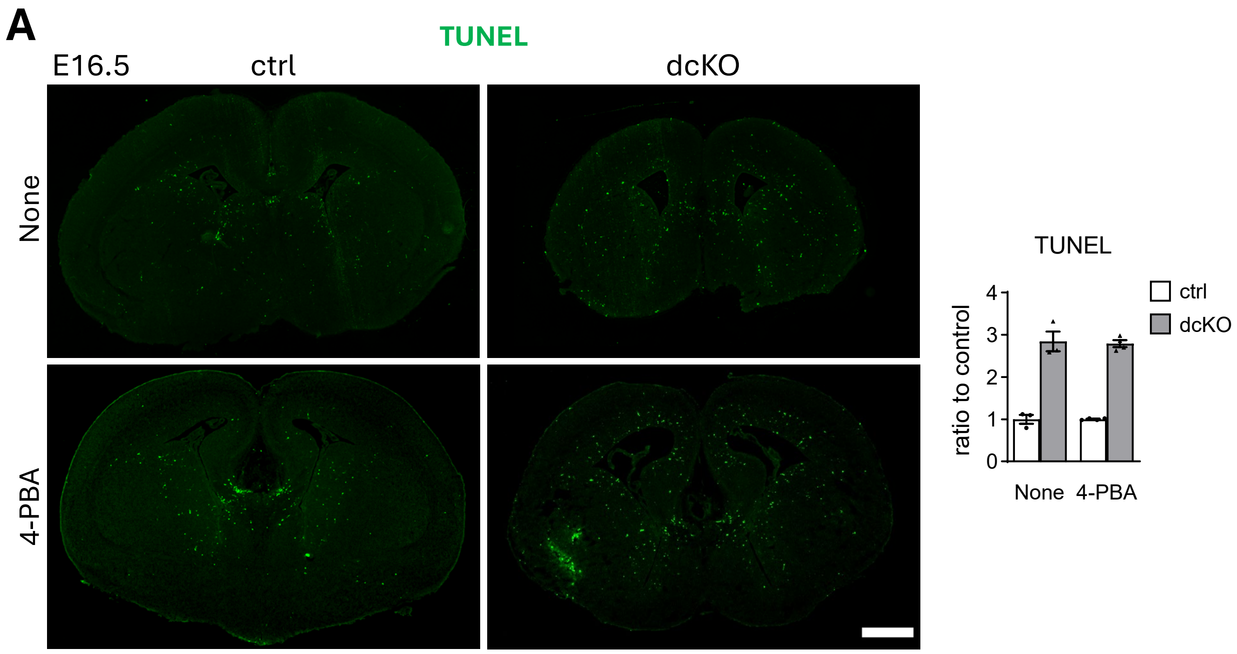
A
TUNEL
E16.5
ctrl
dcKO
None
4-PBA
TUNEL
ratio to control
0
1
2
3
4
None
4-PBA
ctrl
dcKO

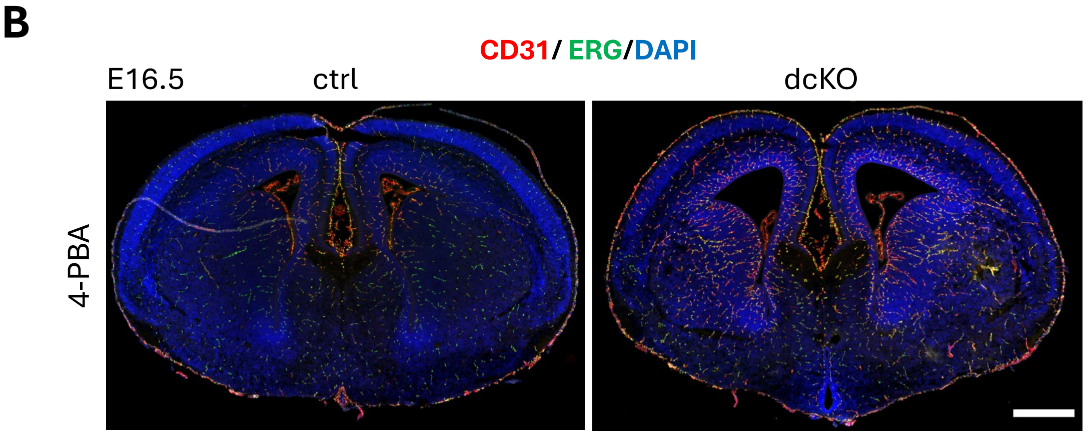
B
CD31/ ERG/DAPI
E16.5
ctrl
dcKO
4-PBA

Figure.S7
